# Structural characterization of surface immobilized platinum hydrides by sensitivity-enhanced ^195^Pt solid state NMR spectroscopy and DFT calculations†

**DOI:** 10.1039/d4sc06450j

**Published:** 2024-11-20

**Authors:** Benjamin A. Atterberry, Erik J. Wimmer, Sina Klostermann, Wolfgang Frey, Johannes Kästner, Deven P. Estes, Aaron J. Rossini

**Affiliations:** a Iowa State University, Department of Chemistry Ames IA 50011 USA arossini@iastate.edu deven.estes@itc.uni-stuttgart.de; b US DOE Ames National Laboratory Ames Iowa 50011 USA; c University of Stuttgart, Department of Chemistry Stuttgart Baden-Württemberg 70569 Germany

## Abstract

Supported single-site platinum hydride compounds are promising heterogeneous catalysts for organic transformations. Few methods exist to describe the structures of single-site Pt catalysts with atomic resolution because of their disordered structures and low Pt loadings. Here, we study the compounds formed when bis(tri-*tert*-butylphosphino)platinum, Pt(P^*t*^Bu_3_)_2_, is supported on dehydroxylated SiO_2_ or SiO_2_–Al_2_O_3_. First, we obtain magic angle spinning (MAS) ^1^H, ^31^P and ^195^Pt ssNMR spectra of four model Pt phosphine compounds with oxidation states of 0 or +2 and coordination numbers between 2 and 4. These compounds are analogs of potential structures present in the supported compounds. MAS ^195^Pt ssNMR spectra were obtained using ^31^P{^195^Pt} sideband selective *J*-resolved and *J*-HMQC experiments. The measured ^1^H and ^31^P chemical shifts, ^31^P–^195^Pt *J*-couplings and ^195^Pt chemical shift (CS) tensors are shown to be diagnostic of oxidation state and coordination number. Room temperature ^1^H ssNMR spectra of Pt(P^*t*^Bu_3_)_2_ supported on SiO_2_ or SiO_2_–Al_2_O_3_ show diagnostic hydride NMR signals, suggesting that Pt(P^*t*^Bu_3_)_2_ undergoes oxidative addition, resulting in surface hydrides and Pt–oxygen bonds to the support surface. MAS dynamic nuclear polarization (DNP) enables ^31^P{^195^Pt} correlation NMR experiments on the supported compounds. These experiments enable the measurement of the ^31^P–^195^Pt *J*-coupling constants and ^195^Pt CS tensors. Combined NMR and DFT analyses suggest that the primary surface platinum species are [HPt(P^*t*^Bu_3_)_2_OSi] on SiO_2_ and [HPt(P^*t*^Bu_3_)_2_]^+^[Si–O^−^–Al] on SiO_2_–Al_2_O_3_. The Pt–oxygen bond length is dependent on the support and estimated as 2.1–2.3 Å and 2.7–3.0 Å for SiO_2_ and SiO_2_–Al_2_O_3_, respectively.

## Abstract 2

^1^H, ^31^P and ^195^Pt solid-state NMR spectroscopy were used to determine the structure of compounds formed by supporting bis(tri-*tert*-butylphosphino)platinum, Pt(P^*t*^Bu_3_)_2_, on dehydroxylated SiO_2_ or SiO_2_–Al_2_O_3_.

## Graphical abstract

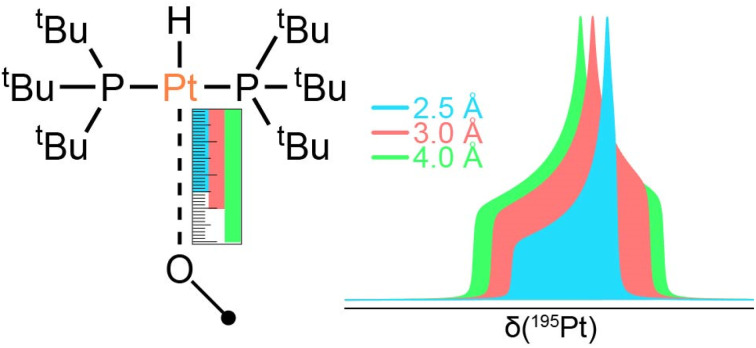

## Introduction

Single-site or single-atom platinum catalysts find widespread application in industrial processes such as fuel production, automotive catalytic converters for emissions control, hydrogenation reactions, hydrosilylation and other organic reactions.^1–6^ One such notable class are the platinum hydride catalysts, renowned for their exceptional catalytic performance in cycloisomerization, isomerization and hydroformylation reactions.^7–9^ However, Pt and Pd catalysts often suffer from a significant drawback in homogeneous catalysis: deactivation through dimerization.^10–12^ To overcome this limitation, heterogeneous catalysts can be used because they prevent dimerization and enable the reusability of the platinum hydride.^13^ Heterogeneous catalysts can be synthesized *via* surface organometallic chemistry (SOMC), which involves immobilizing well-defined metal complexes on metal oxide surfaces.^14–18^ In SOMC, metal oxide samples undergo controlled dehydroxylation at high temperature and high vacuum, resulting in a surface covered with a controlled density of chemically similar OH groups. These OH groups serve as ligands for organometallic complexes, typically achieved through protonolysis reactions where a basic ligand deprotonates the OH group. This process forms a new M–O bond directly to the surface while releasing HX.^19^

Recently, some of the authors of this paper utilized surface OH groups on Brønsted acidic supports like SiO_2_–Al_2_O_3_ to immobilize Pt(PR_3_)_2_ complexes.^13^ Through solid-state nuclear magnetic resonance (ssNMR) spectroscopy, infrared spectroscopy (IR), and X-ray absorption spectroscopy (XAS) characterization, we discovered that the immobilization occurred *via* the apparent oxidative addition of OH groups to the Pt(0) center in the precursor, resulting in Pt(ii)–H species with a new Pt–O bond to the surface. This finding is quite rare, with only two known examples reported in the literature so far.^13,20^ The Pt–O bond length (2.01 Å) measured by EXAFS in our study was indicative of a four-coordinate Pt–H with a slightly elongated Pt–O bond.^21^ However, the Pt–H ^1^H chemical shift (−36 ppm) and *J*_Pt–H_ (2400 Hz) values were more consistent with formation of cationic three-coordinate Pt–H, such as [(^*t*^Bu_3_P)_2_Pt–H]^+^. Based on these observations, we proposed that the structure on the surface lies somewhere between three- and four-coordinate, where the nature of the Pt–O bond is something in between a true Pt–O bond and an ion pair. Thus, additional experimental methods are needed to characterize the structure of these surface species.

^195^Pt ssNMR spectroscopy is potentially an appealing method to investigate the structure of surface-supported platinum organometallics. Essential information about the chemical and electronic environments of Pt centers in catalysts are encoded in the ^195^Pt chemical shift (CS) tensor, which is sensitive to the bonding character and symmetry of the neighboring ligands.^22–32^ However, ^195^Pt NMR experiments in the solid-state are often challenging because Pt compounds often exhibit a large ^195^Pt chemical shift anisotropy (CSA) on the order of several thousand parts-per-million (ppm), especially for Pt(ii) complexes, which typically adopt a square planar geometry.^23,33,34^ Several notable methods have been proposed to expedite the measurement of ^195^Pt ssNMR spectra and CS tensors. Schurko and coworkers have incorporated Wideband Uniform Rate Smooth Truncation (WURST) pulses^35,36^ and broadband adiabatic inversion cross-polarization (BRAIN-CP)^37^ into Carr–Purcell–Meiboom–Gill (CPMG)^38^ sequences that allow static ^195^Pt ssNMR spectra to be acquired with increased sensitivity on molecular complexes. Unfortunately, it is time-consuming to study single-site Pt catalysts using these methods because they have low Pt loadings (<5 wt%). To address this challenge, dynamic nuclear polarization surface enhanced NMR spectroscopy (DNP SENS) has been used to increase the sensitivity of static^37,39^ and magic angle spinning (MAS)^27,40^^195^Pt ssNMR experiments. We have also previously shown that a sensitive spy nucleus like ^1^H, ^31^P, or ^13^C can be used to enable acquisition of wideline ^195^Pt ssNMR spectra, including for detection of surface species.^27,41,42^ Further gains in sensitivity have been achieved by combining indirect detection MAS experiments with DNP SENS. Most indirect detection ssNMR experiments have used MAS frequencies >25 kHz, while slower MAS frequencies less than 12.5 kHz have not been employed yet.^27^

**Fig. 1:**
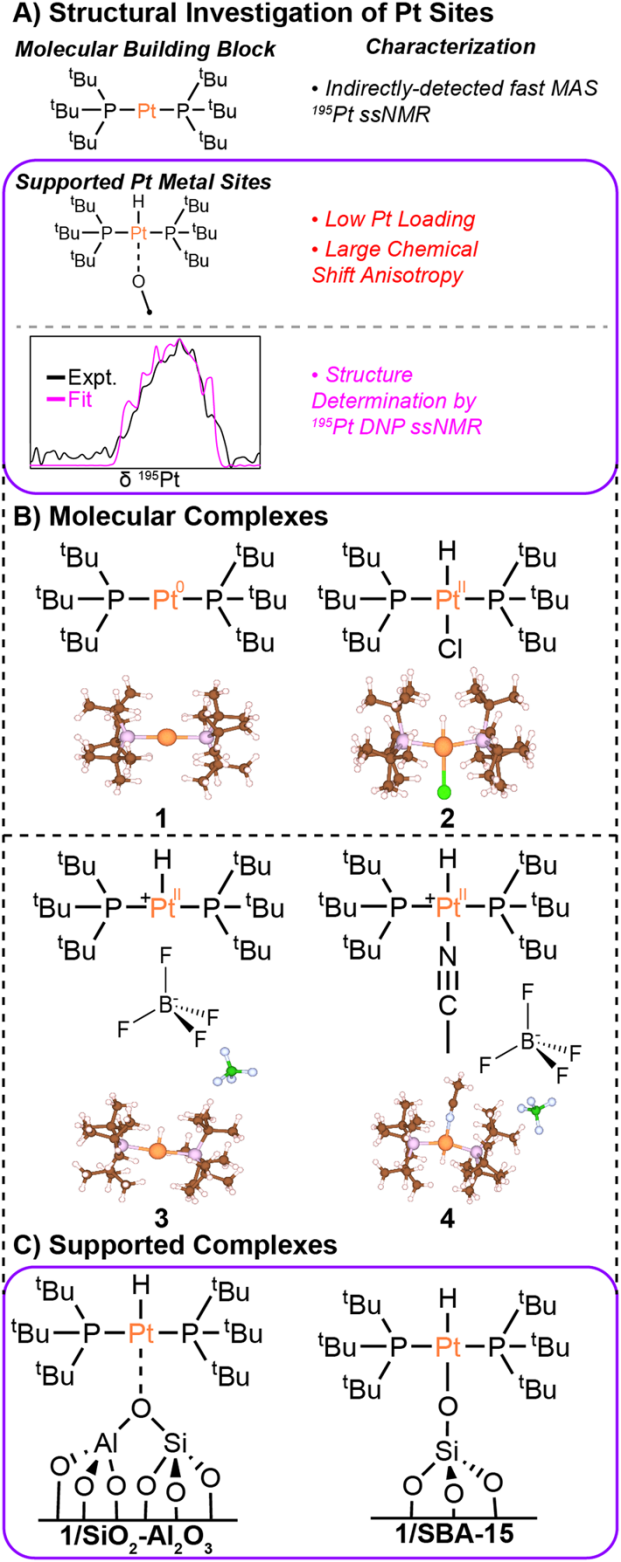
(A) Graphical summary of the characterization of molecular and surface-supported Pt complexes. (B) Molecular structures and X-ray crystal structures are shown for Pt(P^*t*^Bu_3_)_2_ (1), HPt(P^*t*^Bu_3_)_2_Cl (2),^43^ [HPt(P^*t*^Bu_3_)_2_]BF_4_ (3), and [HPt(P^*t*^Bu_3_)_2_NCCH_3_]BF_4_ (4). (C) Hypothesized structures are shown for the supported single-site Pt complexes discussed in this work. The Pt loadings in the supported single-site materials were 2 wt% (1/SiO_2_–Al_2_O_3_) and 1.9 wt% (1/SBA-15).

In this work, we use room temperature fast MAS ^1^H{^195^Pt} and ^1^H–^31^P{^195^Pt} *J*-resolved and *J*-HMQC experiments to investigate four different molecular bis(tri-*tert*-butylphosphino)platinum compounds (Fig. 1). These compounds feature Pt in oxidation states of 0 and +2 and some of the compounds have coordinated hydrides. We then use slow MAS (12.5 kHz) cryogenic DNP SENS ^1^H–^31^P{^195^Pt} *J*-resolved experiments to study two low Pt wt% (1.9 and 2 wt%) single-site catalysts. By comparing the ^1^H, ^31^P, and ^195^Pt ssNMR spectra of two-, three-, and four-coordinate Pt model complexes (1–4) with those of the surface complexes, we were able to gain insights into the nature of the Pt–O bond on the surface. These methods, combined with DFT calculations, offer a blueprint of the electronic and coordination spheres of the molecular and surface-supported complexes. Overall, these experiments allow us to put forward evidence-based structural models of surface-supported platinum hydride compounds.

## Results & discussion

### Overview

First, we applied ssNMR spectroscopy to molecular platinum hydride and phosphine compounds (Fig. 1). These compounds serve as analogs for different surface species that could be present when supporting bis(tri-*tert*-butylphosphino)platinum (1) on SBA-15 or SiO_2_–Al_2_O_3_. For each molecular compound, we measured the ^1^H, ^31^P, and ^195^Pt isotropic chemical shifts, ^195^Pt anisotropic chemical shift tensor parameters, and ^31^P–^195^Pt and ^1^H–^195^Pt *J*-couplings (when possible). These measured NMR parameters are diagnostic of the Pt oxidation state and coordination environment. These NMR parameters are also reproduced with DFT calculations. In the last part of the paper, we use DNP-enhanced ^31^P detected ssNMR experiments to study the immobilized platinum phosphine compounds supported on SBA-15 and on SiO_2_–Al_2_O_3_. Comparison of experimental and calculated ^195^Pt CS tensor parameters and hydride ^1^H chemical shifts are used to elucidate structural models for the surface species formed on the two different supports.

### ^1^H, ^31^P and ^195^Pt ssNMR experiments on molecular complexes

**Fig. 2:**
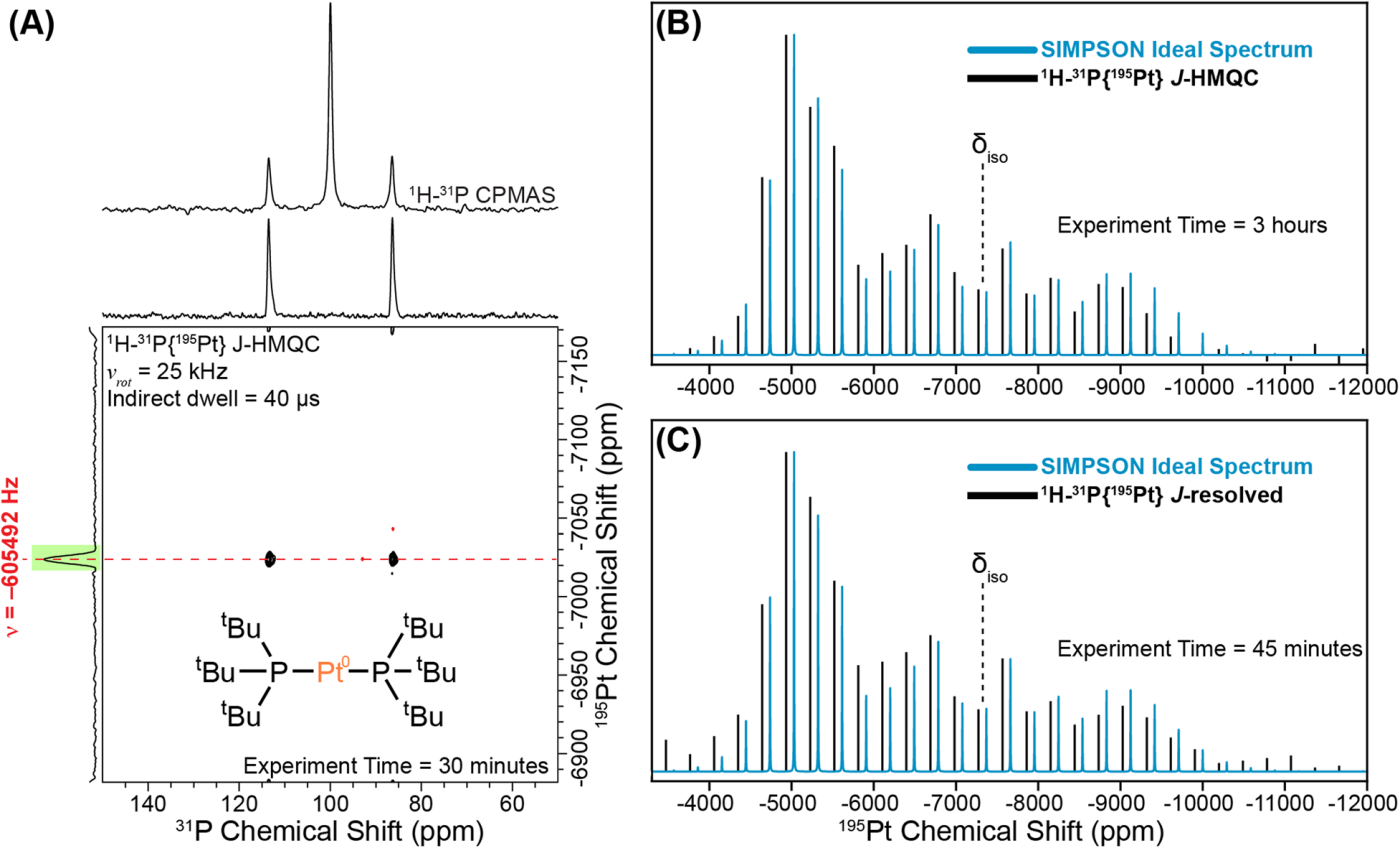
Graphical illustration of implementing a sideband selective experiment for compound 1. (A) 1D ^1^H–^31^P CPMAS spectrum overlaid on the rotor-synchronized ^1^H–^31^P{^195^Pt} *J*-HMQC spectrum. Reconstructed ^195^Pt ssNMR spectra obtained using (B) sideband selective ^1^H–^31^P{^195^Pt} *J*-HMQC and (C) ^1^H–^31^P{^195^Pt} *J*-resolved experiments (black lines). The intensities of the sidebands are compared to the SIMPSON-calculated ideal ^195^Pt MAS NMR spectrum (blue lines). All NMR experiments were performed with a 25 kHz MAS frequency and a magnetic field of 9.4 T. The ^195^Pt saturation pulses were 80 μs in duration with RF fields of 5 kHz and 9 kHz RF for *J*-HMQC and *J*-resolved, respectively.

We begin with a study of the molecular platinum phosphine compounds, 1–4. Fig. 2A shows the ^31^P CPMAS NMR spectrum and a rotor-synchronized ^31^P{^195^Pt} *J*-HMQC spectrum of 1. The 1D ^31^P CPMAS NMR spectrum shows two satellites that arise from a *J*-coupling of 4407 Hz between ^195^Pt and ^31^P. In the 2D *J*-HMQC spectrum, only the satellite peaks are visible. The ^31^P{^195^Pt} *J*-HMQC was recorded with the *t*_1_-increment set to a rotor period (40 μs), corresponding to the indirect dimension spectral width being equal to the MAS frequency (25 kHz). Rotor synchronization of the indirect dimension maximizes sensitivity of the 2D experiments because all the spinning sidebands will be aliased onto a single peak. From the 2D ^31^P{^195^Pt} *J*-HMQC spectrum, we can determine the offset of a single ^195^Pt spinning sideband, which was −605492 Hz from the reference ^195^Pt frequency in this case. Once we know the offset of a single sideband, we can then perform sideband selective 1D ^31^P{^195^Pt} *J*-HMQC or *J*-resolved NMR experiments that enable us to map out the intensity of each spinning sideband that makes up the entire ^195^Pt MAS ssNMR spectrum (Fig. 2B and C).^41,42^ The sideband selective NMR experiments use ^195^Pt selective long (SL) pulses that have durations of one rotor cycle or longer and RF fields less than 50 kHz.^44^ Each line represents the measured intensity or dephasing of the ^31^P NMR signal at the indicated ^195^Pt offset. Note that sideband selective experiments also require the RF-field strength of the pulses to be accurately calibrated because using an RF field 10 kHz higher-than-optimal will result in an NMR spectrum that does not accurately reconstruct the MAS ^195^Pt ssNMR spectrum.^42^

Fig. 2B and C show experimental ^1^H–^31^P{^195^Pt} *J*-HMQC and *J*-resolved sideband selective NMR spectra of 1 obtained with 80 μs ^195^Pt saturation pulses, with RF fields of 5 kHz and 9 kHz RF for *J*-HMQC and *J*-resolved, respectively.^42^ In both cases, the sideband selective spectra match closely with the overlaid ideal MAS ^195^Pt ssNMR spectrum. The NMR spectra shown in Fig. 2B and C were obtained in 3 hours and 45 minutes, respectively.

**Fig. 3:**
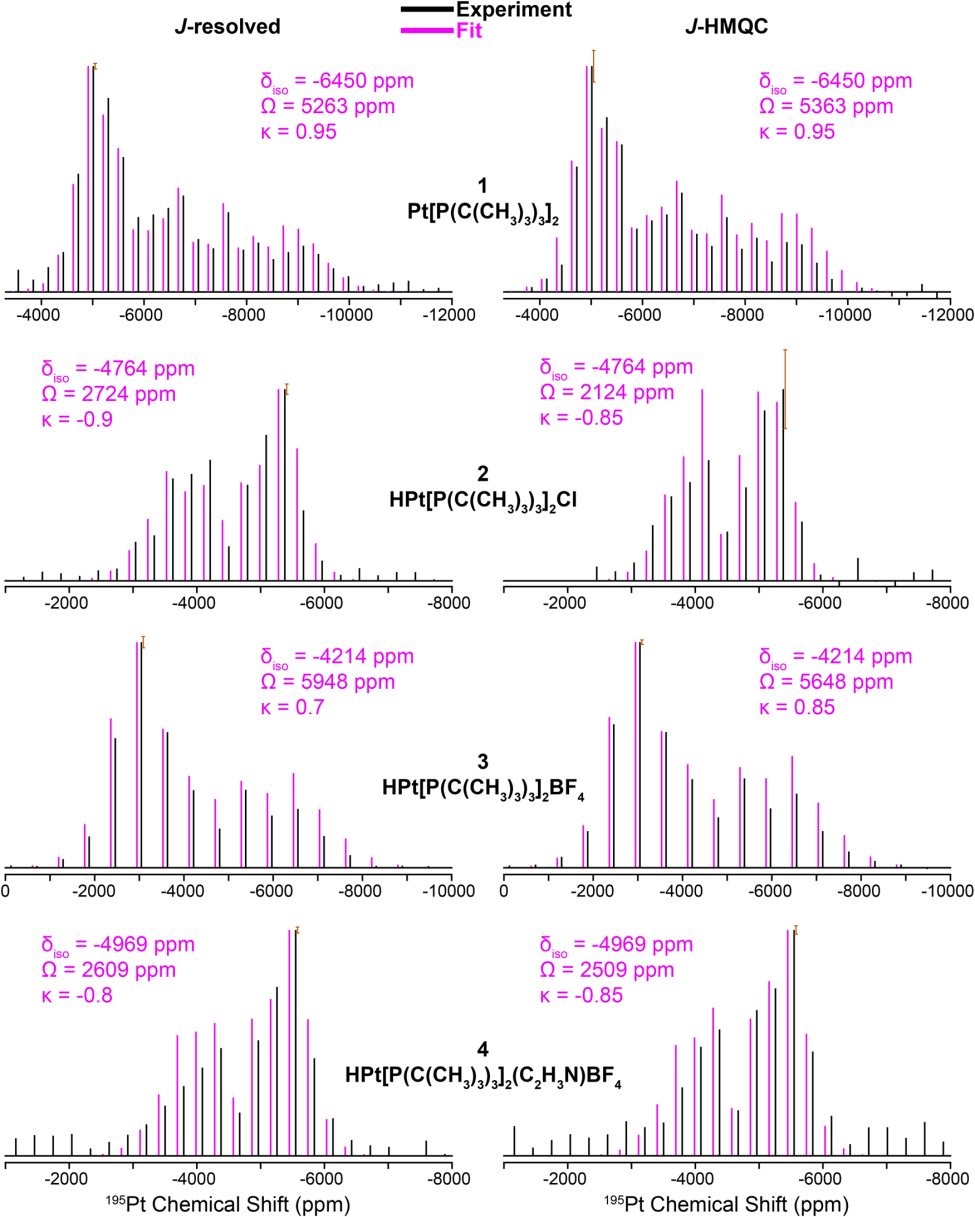
^195^Pt ssNMR spectra reconstructed using ^31^P{^195^Pt} *J*-resolved (left) and ^31^P{^195^Pt} *J*-HMQC (right) sideband selective experiments along with SIMPSON fits for structures 1, 2, and 4. ^1^H{^195^Pt} *J*-resolved and *J*-HMQC experiments are shown for 3. Sideband selective experiments and SIMPSON fits are colored black and pink, respectively. The chemical shift (CS) tensors from the SIMPSON fits are given with the Herzfeld–Berger convention.^45^ Error bars are overlaid on the most intense spinning sidebands.

Fig. 3 shows ^31^P{^195^Pt} *J*-resolved and *J*-HMQC sideband selective experiments for 1, 2, and 4 obtained with a 25 kHz MAS frequency. For 3, ^1^H{^195^Pt} *J*-resolved and *J*-HMQC sideband selective experiments were performed at 50 kHz MAS. ^1^H spin echo and ^1^H–^31^P CPMAS NMR spectra for complexes 1–4 are shown in Fig. S1.† Overall, Fig. 3 illustrates that we can quickly and accurately measure the ^195^Pt ssNMR spectra of Pt–phosphine or hydride compounds. Below, we discuss trends in the measured ^195^Pt, ^1^H and ^31^P chemical shifts and *J*-coupling constants.

The clear identification of oxidation state and coordination sphere symmetry can be achieved by analyzing the trends in the ^195^Pt CS tensors. Comparing the ^195^Pt isotropic shifts reveals that complex 1 with an oxidation state of Pt(0) gives rise to the most negative shift (−6450 ppm) and complexes 2–4 with Pt(II) have more positive shifts (−4200 ppm to −5000 ppm). This observation is consistent with the results obtained from ^195^Pt solution NMR experiments.^46–48^ The second parameter that gives insight about geometry at the Pt atoms is the span (*Ω*). As shown in Fig. 3, *Ω* is much larger for the three-coordinate complex (3, *ca.* 5750 ppm) in comparison to the four-coordinate complexes (2, 4, *ca.* 2500 ppm). Although, we caution that the *Ω* is also sensitive to the electronic nature of the ligands, with stronger donors leading to smaller Ω.^41^ The last parameter to discuss would be the asymmetry parameter of the CS tensor (*κ*). For the four-coordinate compounds, *κ* is close to −1, indicating the presence of a mirror plane (or *C*_2_ symmetric or higher rotational axis) and the requirement for two of the three parameters in the CS tensor to be the same. Similarly, the three- and two-coordinate compounds exhibit a *κ* close to +1. The transition from *κ* = −1 to *κ* = +1 can be attributed to shifts in the electronic configuration due to differences in the coordination number (less electron donation perpendicular to the P–Pt–P axis), consequently altering the energies of the d orbitals and thus the positions of the corresponding tensor components. The ^195^Pt CS tensor parameters provide a reliable means of determining the oxidation state or coordination environment of an unknown complex.

In addition, the ^31^P and ^1^H ssNMR spectra are diagnostic of the Pt oxidation state. Specifically, a three-coordinate Pt–H complex exhibits a characteristic hydride chemical shift in the ^1^H NMR shift at *ca.* −38 ppm, whereas the four-coordinate complexes display shifts around −19 ppm (Fig. S1†). We also observed that the ^31^P shift for 1 is centered at 100 ppm (Pt^0^) and 2–4 have ^31^P shifts around 70–80 ppm (Pt^II^). Finally, the ^31^P–^195^Pt *J*-couplings could be directly measured based on the difference in frequencies of the satellite peaks in the ^31^P NMR spectra, except for 4, for which we used a *J*-resolved evolution curve to measure the *J*-coupling (Fig. S2†). For complex 1, a sizeable ^31^P–^195^Pt *J*-coupling is observed (^1^*J*(^31^P–^195^Pt) = 4407 Hz), which is typical of Pt^0^ compounds.^49^ But for complexes 2–4, ^1^*J*(^31^P–^195^Pt) decreases to ∼2500 to ∼3000 Hz. The ^1^H–^195^Pt *J*-couplings for complexes 2–4 were also measured and ^1^*J*(^1^H–^195^Pt) is ∼1000 Hz for 4-coordinate complexes (2 & 4) and 2630 Hz for the 3-coordinate complex (3). These characteristic chemical shifts and coupling constants supply valuable information that can aid in the investigation of surface-supported samples. The root mean square error plots for the numerical fits of the ^31^P{^195^Pt} *J*-resolved and *J*-HMQC sideband selective experiments on 1–4 are shown in Fig. S3.†

### DFT calculations of NMR observables

**Fig. 4:**
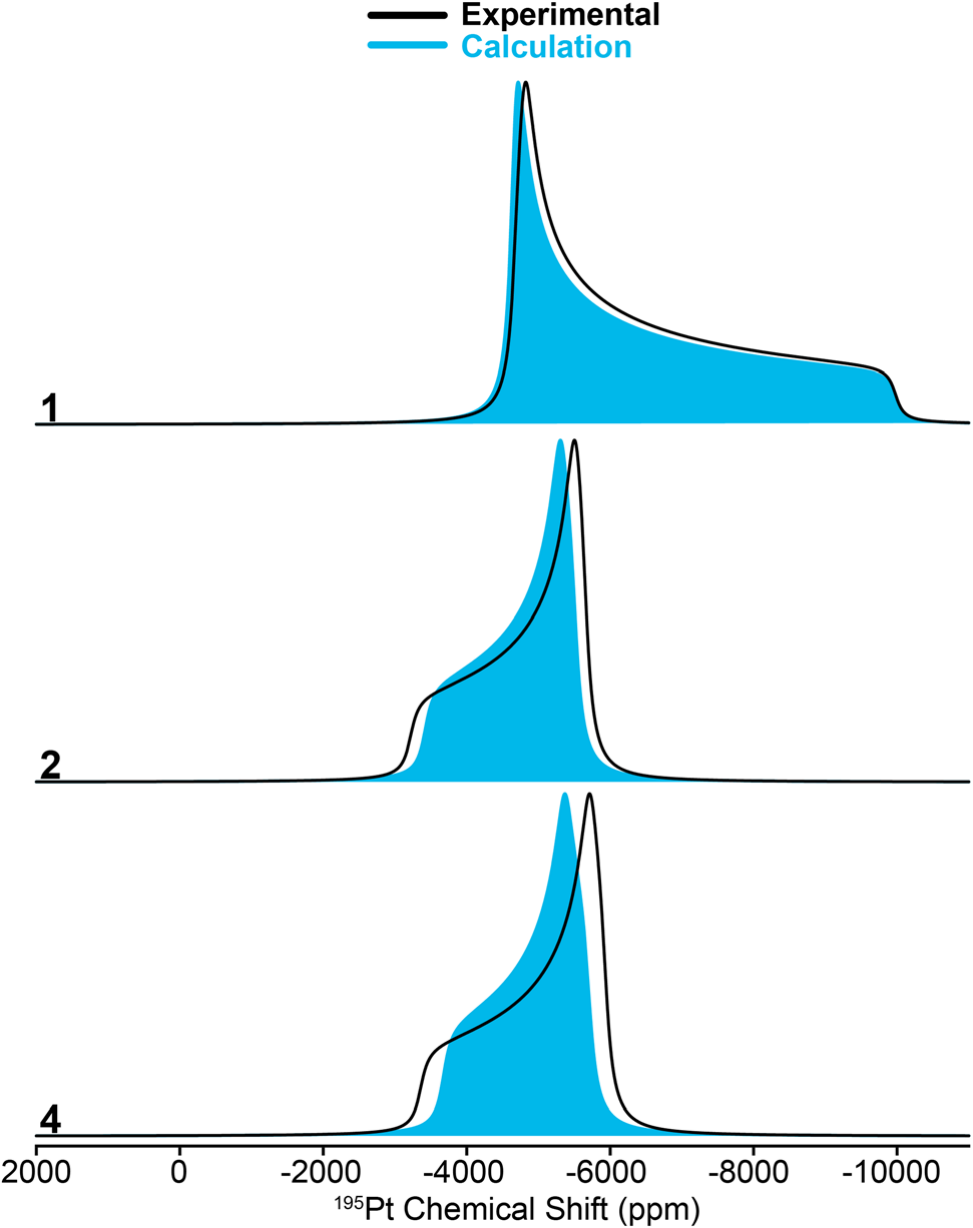
Simulated ^195^Pt static ssNMR spectra for compounds 1, 2, and 4. Black lines show the spectra simulated using ^195^Pt CS tensors measured with the MAS ssNMR experiments and solid blue patterns correspond to DFT calculated ^195^Pt CS tensors.

Modern computational chemistry methods can accurately predict CS tensors^50,51^ and *J*-couplings^52,53^ for heavy nuclei such as ^195^Pt. We geometrically optimized the H-atom positions in the X-ray crystal structures of complexes 1–4 using DFT calculations in CASTEP^54^ and then used molecular DFT calculations in the Amsterdam Modeling Suite^55^ along with a hybrid density functional^56,57^ to predict the NMR parameters. Overall, the calculated and experimental ^1^H, ^31^P and ^195^Pt NMR parameters are in good agreement for complexes 1, 2, and 4 (Table 1 and Fig. 4).

**Table 1:** Experimental and calculated chemical shifts and spin–spin couplings

Compound	Pt oxidation state	Geometry	Method	Hydride δ_iso_ (^1^H) (ppm)	δ_iso_ (^31^P) (ppm)	^*1*^ *J* (^31^P-^195^Pt) (Hz)	^*1*^ *J* (^1^H-^195^Pt) (Hz)	^195^Pt δ_iso_ (ppm)	^195^Pt Ω (ppm)	^195^Pt κ
1	+0	Linear	Experiment	—	100	4407	—	−6450	5313	0.95
			X-ray structure	—	100	5226	—	−6392	5272	0.99
2	+2	Square planar	Experiment	−19	76	3025	1100	−4764	2424	−0.88
			X-ray structure	−17	81	3386	1071	−4716	2078	−0.81
3	+2	T-shaped	Experiment	−38	88	2624	2630	−4214	5798	0.78
			X-ray structure	−35	92	3088	3117	−3717	7325	−0.10
			One trans	−27	62, 34	2114	2355	−4154	5168	−0.17
			Two orthogonal	−32	62, 47	3031	4278	−3188	7109	0.33
4	+2	Square planar	Experiment	−19	95, 78	2793	1030	−4969	2559	−0.83
			X-ray structure	−15	90, 66	2822, 3049	1114	−4885	2019	−0.67
1/SiO_2_–Al_2_O_3_	+2	Square planar/T-shaped	Experiment	−35	82	2433	2400	−4841	5680	−0.6
			Optimized structure	−32	85	3434	1882	−4385	6314	−0.3
1/SBA-15	+2	Square planar/T-shaped	Experiment (hydride)	−26	72	2908	1034	−4441	4080	0.0
			Experiment (C–H activated)	−	52	—	—	−3541	7980	0.0
			Optimized Structure	−26	99	3315	1213	−4484	3775	−0.8

**Fig. 5:**
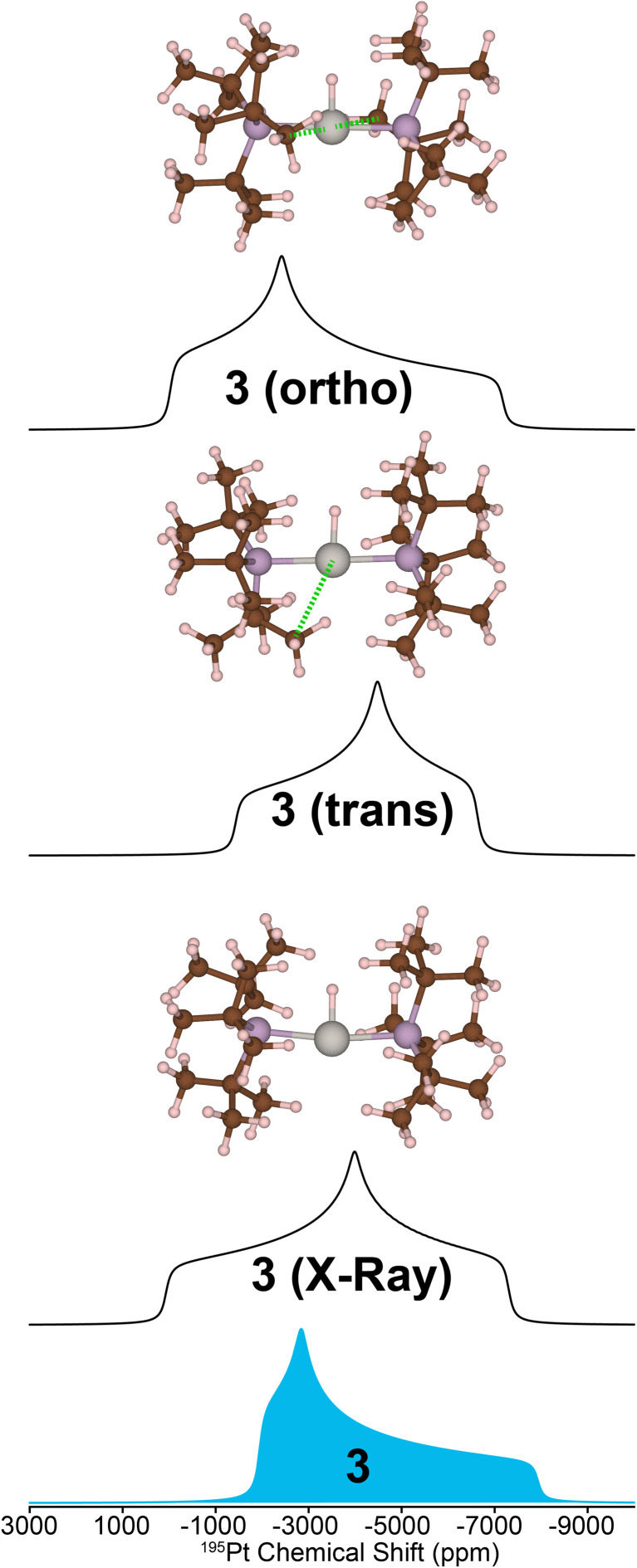
Comparison of the ^195^Pt ssNMR spectrum for complex 3 and the DFT-calculated ^195^Pt NMR spectra for different structural models. Calculations were performed with the single crystal X-ray diffraction structure (bottom), a modified structure featuring one agostic interaction between Pt and a methyl group C–H bond that was oriented *trans* to the platinum hydride bond (middle), and two Pt methyl C–H agostic interactions orthogonal to the platinum hydride bond (top). A green dashed line indicates an agostic interaction.

While calculating the CS tensors with optimized H atom positions and crystallographic coordinates for heavy atoms (crystal structures for 2–4 are shown in Fig. S13–S15 and Tables S3–S5†) worked well for complexes 1, 2, and 4, the agreement for complex 3 was less than satisfactory. As for 1, 2, and 4 we calculated the ^195^Pt CS tensor using the single crystal X-ray diffraction structure (with DFT optimization of the H atom positions). This calculation gave a predicted ^195^Pt NMR spectrum with *δ*_iso_ = −3717 ppm, *Ω* = 7325 ppm, and *κ* = −0.10, which was significantly different from the experimental spectrum that we measured for 3 (*δ*_iso_ = −4214 ppm, *Ω* = 5798 ppm, and *κ* = 0.78) (Fig. 5, bottom). Examination of the experimental ^1^H and ^31^P NMR spectra shows that the complex has not decomposed before measurement (signals for Pt–H and of the P^*t*^Bu_3_ groups are correct, Table 1 and Fig. S1†). However, we have observed that the crystal lattice of 3 is not stable over long time periods due to slow loss of the dichloromethane solvents of crystallization from the crystal lattice (Fig. S4†). Indeed, placing the crystals under vacuum even for short periods results in reduced crystallinity. We theorized that the local structure of 3 could change upon loss of the solvents of crystallization to give a different coordination environment – changing the CS tensor. One commonly observed interaction in similar T-shaped cationic Pt phosphine complexes is the formation of C–H agostic interactions, which is notably absent from the crystal structure of complex 3.^58–62^ Indeed, DFT calculations showed that optimization of the methyl positions resulted in a new structure, 3(*trans*), which features a C–H agostic interaction in the position *trans* to the hydride ligand is *ca.* 4.5 kcal mol^−1^ more stable than the structure with no agostic interactions (3(X-ray)). The optimized structure of 3(*trans*) showed a Pt–C distance of 2.77 Å and a ∠H–Pt–C = 157.30°, both of which are typical for such complexes.^63^ We therefore calculated the ^195^Pt NMR parameters of 3(*trans*) and found that the agreement with our experimental observations was now significantly better (*δ*_iso_ = −4154 ppm, *Ω* = 5168 ppm, and *κ* = −0.17). It is evident that if methyl groups participate in secondary bonding there is a change two of the principal components of the ^195^Pt CS tensor (*δ*_11_ and *δ*_22_) but not *δ*_33_. Still, the agreement between the calculated NMR parameters of 3(*trans*) and the experimental spectrum of 3 is not perfect. The *δ*_22_ tensor component shows the largest deviation, as demonstrated by the very different *κ* value for 3(*trans*). We also examined another model, 3(ortho), having agostic interactions in the apical position (orthogonal to the H–Pt–P plane). This led to a structure with two agostic interactions perpendicular to the T-shaped coordination structure and had an energy that is *ca.* 40 kcal mol^−1^ higher than 3(*trans*). The NMR parameters of this species were also quite different than the experimental spectrum. Calculations predict a large ^1^H–^195^Pt *J*-coupling for 3(ortho) (3031 Hz), which is much larger than what is predicted for 3(*trans*) (2114 Hz) and what is observed experimentally (2630 Hz). The ^31^P and ^1^H spectra predicted for 3(*trans*) also match the experimental spectrum better than what is seen for the other two models. However, we only see one chemically equivalent peak for the P^*t*^Bu_3_ groups in 3, rather than the two inequivalent signals predicted for 3(*trans*). Therefore, we hypothesize that the experimental spectrum of 3 can be explained by formation of a *trans*-agostic interaction upon loss of solvated dichloromethane from the crystal lattice. The observed chemical equivalency of the P atoms of the two phosphines of 3 could be the result of rapid exchange between methyl groups in the agostic position.

In summary, NMR experiments on complexes 1–4 illustrate how ^195^Pt CS tensors can be measured and related back to the structural features and Pt oxidation states of the compounds. The ^31^P and ^1^H chemical shifts and ^31^P–^195^Pt *J*-coupling constants also provide valuable structural information. Using relativistic DFT calculations it is possible to accurately reproduce the NMR observables. With these capabilities in hand, we now turn to the structural characterization of surface-supported platinum phosphine compounds.

### ssNMR experiments on surface-supported complexes

**Fig. 6:**
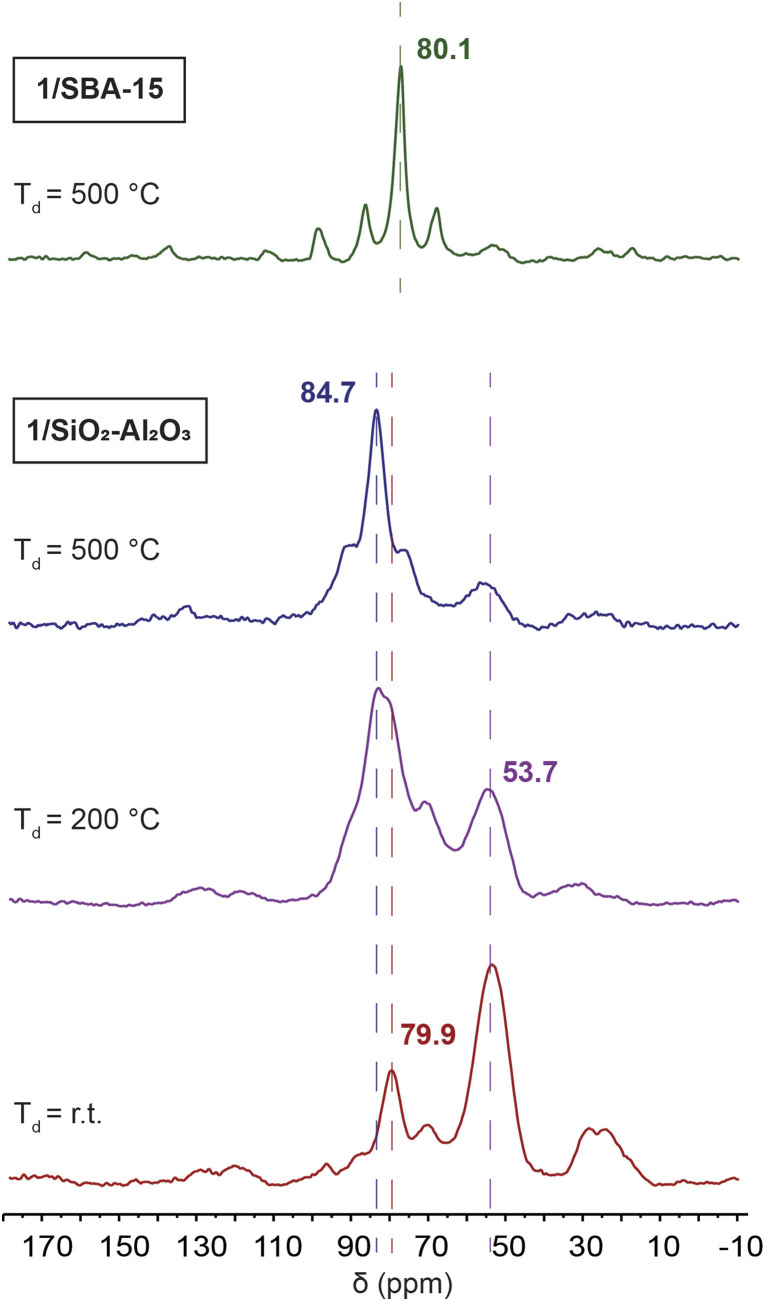
Room temperature ^31^P CPMAS ssNMR spectra of 1/SBA-15 (green) and 1/SiO_2_–Al_2_O_3_ made with SiO_2_–Al_2_O_3_ had not been dehydroxylated (red), dehydroxylated at *T*_d_ = 200 °C (purple), and dehydroxylated at *T*_d_ = 500 °C (blue).

We sought to understand the chemical structure of the surface-supported complexes 1/SiO_2_–Al_2_O_3_ and 1/SBA-15. The surface immobilized complexes were synthesized by reacting a solution of 1 at room temperature with either SiO_2_–Al_2_O_3_ or SBA-15 for 18 hours, as we reported previously.^13^^31^P CPMAS NMR spectra show that the results of the immobilization are highly dependent on the level of dehydroxylation of the material (Fig. 6).

We immobilized 1 on SiO_2_–Al_2_O_3_ that had not been dehydroxylated, as well as material dehydroxylated at 200 °C and at 500 °C. When the material had not been dehydroxylated, a major peak at 53.7 ppm corresponding to the protonated phosphine HP^*t*^Bu_3_^+^ and a minor peak at 79.9 ppm corresponding to the four coordinate hydrated complex [HPt(P^*t*^Bu_3_)_2_(OH_2_)]^+^ were observed.^64,65^ Additionally, two other minor peaks can be seen at 70 ppm and 24 ppm that have been more difficult to assign. Four coordinate platinum hydrides are known to activate the C–H bonds of ligated P^*t*^Bu_3_ at relatively mild conditions to give the cyclometallated Pt complexes and loss of H_2_ (*vide infra*).^11^ We did not observe formation of tri-*tert*-butylphosphine oxide (66.5 ppm)^66^ in any of the grafted samples. However, the chemical shift of tri-*tert*-butylphosphine oxide can vary considerably due to hydrogen bonding to surface Brønsted and Lewis acid sites.^67–71^

**Scheme 1:**
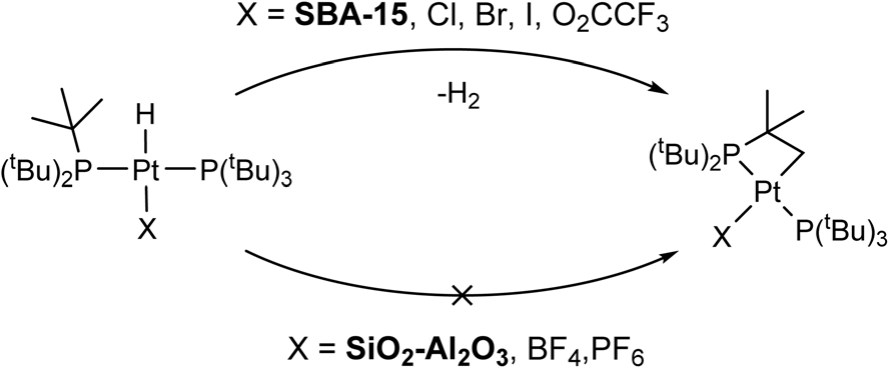
C–H activation and formation of cyclometallated Pt species on silica surfaces (top) and non-activated 3-coordinated Pt–H on SiO_2_–Al_2_O_3_ surfaces (bottom).

The rate of C–H activation is highly dependent on the identity of the fourth ligand. For four-coordinated Pt complexes, the metalation rate decreases in the order I > Br > Cl > O_2_CCF_3_ ≈ NO_3_, with the *trans*–I complex undergoing cyclometallation in less than 24 hours.^11^ In contrast, for three-coordinated complexes with non-coordinating anions (*e.g.* BF_4_^−^), no cyclometallation is observed (Scheme 1).^64^ While the mechanism of the C–H activation in these cases is not completely clear, there seems to be a relationship between the rate of cyclometallation and the *trans*-influence of the fourth ligand, which matches the trend observed above. The signals of cyclometallated complexes of the type [Pt(–CH_2_CMe_2_P^*t*^Bu_2_)(P^*t*^Bu_3_)]^+^ in the ^31^P NMR spectrum also vary depending on the identity of the counterion/fourth ligand. The resonance corresponding to coordinated P^*t*^Bu_3_ falls between 70 and 59 ppm while the cyclometallated phosphine can have values between 25 and −16 ppm. Thus, the observed NMR signals at 70 and 24 ppm in 1/SiO_2_–Al_2_O_3_ dehydroxylated at room temperature could be consistent with cyclometallation after the initial preparation (*vide infra*).^11,72^ However, upon dehydroxylation of the surface at temperatures up to 500 °C, all of these signals give way to a complex with spectral signals similar to that of three coordinate 3 (*δ*^31^_P_ = 84.7 ppm, *δ*_Pt–H_ = −35 ppm), which becomes the main species on the surface dehydroxylated at 500 °C. Degradation over time was observed towards forming more protonated phosphine, with only small amounts of C–H activation. Therefore, our subsequent analysis focused on 1/SiO_2_–Al_2_O_3_ prepared with SiO_2_–Al_2_O_3_ that was dehydroxylated at 500 °C. Since we mostly observe the four-coordinate [HPt(P^*t*^Bu_3_)_2_(OH_2_)]^+^ complex on the surface of partly hydroxylated supports, this would explain its propensity for undergoing slow cyclometallation, which is not observed in the three-coordinate [HPt(P^*t*^Bu_3_)_2_]^+^ species formed on the dehydroxylated surfaces.

Interestingly, regardless of the dehydroxylation temperature, only one species forms for 1/SBA-15 indicated by the presence of a ^31^P NMR signal at 80 ppm. However, over time, we also observed C–H activation, which might be accelerated under reduced pressure when the compounds are stored in sealed tubes under vacuum (see Fig. S6–S8†); two additional ^31^P peaks are visible at 56 and 24 ppm in the DNP-enhanced ^31^P ssNMR spectrum of 1/SBA-15. These chemical shifts are consistent with the literature-reported C–H activated Pt(P^*t*^Bu_3_)_2_ complex.^72^

**Fig. 7:**
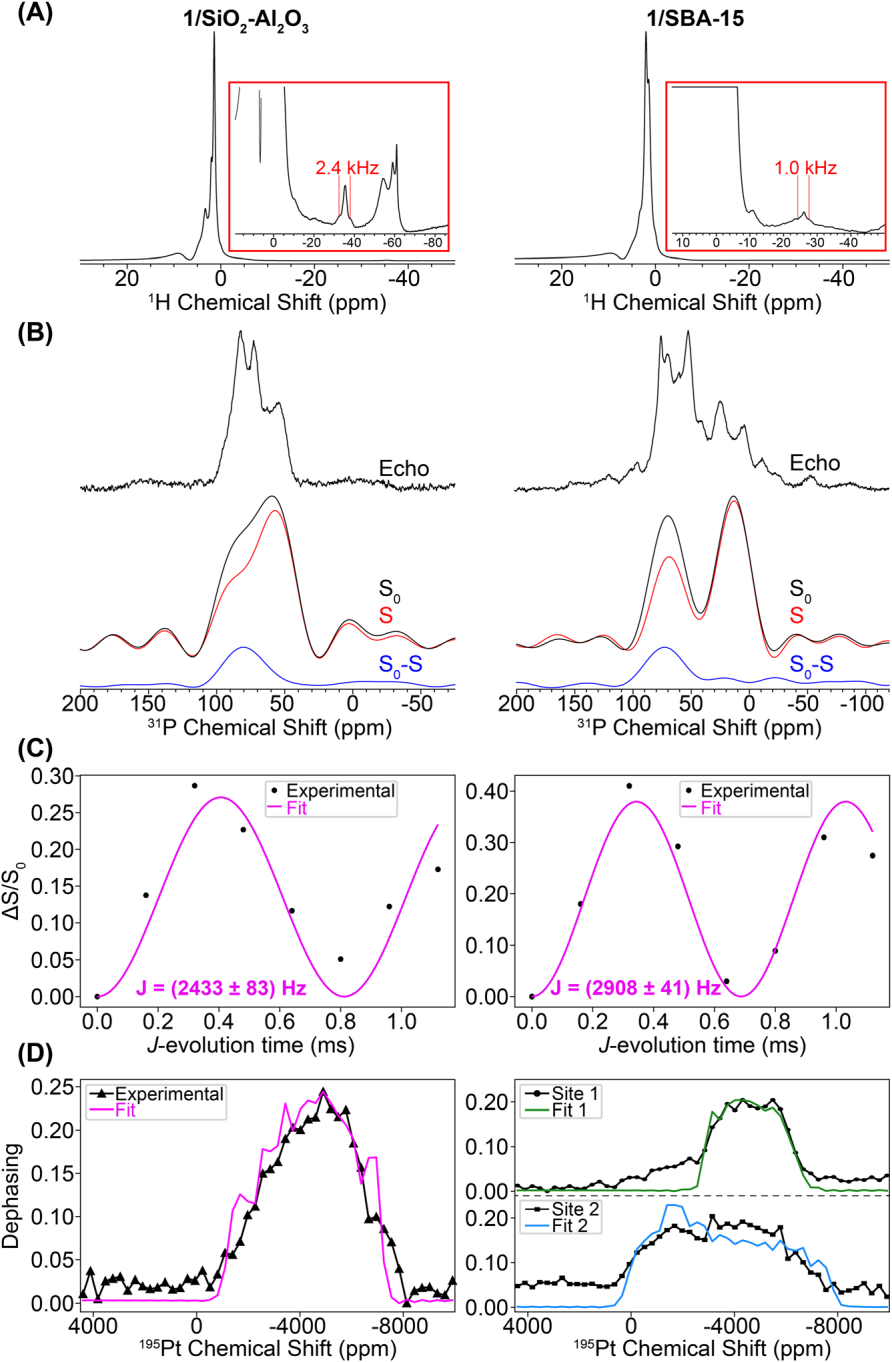
(A) Room temperature MAS ^1^H spin echo NMR spectra of 1/SiO_2_–Al_2_O_3_ (left) and 1/SBA-15 (right). (B) DNP-enhanced ^31^P{^195^Pt} *J*-resolved control (*S*_0_), dephasing (*S*) and difference (*S*_0_ − *S*) spectra. Spectra were obtained with CPMG detection and co-addition of the spin echoes in the time domain. Spectra were acquired with a 0.32 ms *J*-evolution time (spin echo duration). A higher resolution DNP-enhanced ^31^P CPMAS spin echo spectrum is overlaid. (C) ^31^P{^195^Pt} *J*-evolution plots. Black circles and pink lines correspond to experimental data points and least-squares fit, respectively. (D) Plot of ^31^P{^195^Pt} *J*-resolved signal dephasing as a function of ^195^Pt offset. The black lines with data markers correspond to experiments on complexes 1/SiO_2_–Al_2_O_3_ and 1/SBA-15, and the pink line is the SIMPSON fit of the experiment on complex 1/SiO_2_–Al_2_O_3_ (*δ*_iso_ = −4841 ppm, *Ω* = 5680 ppm, *κ* = −0.6). The green and blue lines are the SIMPSON fits for site 1 (*δ*_iso_ = −4441 ppm, *Ω* = 4080 ppm, *κ* = 0.0) and site 2 (*δ*_iso_ = −3541 ppm, *Ω* = 7980 ppm, *κ* = 0.0), respectively, of 1/SBA-15.

We used a combination of room temperature 25 kHz MAS frequency and 100 K DNP SENS NMR experiments (12.5 kHz MAS frequency) to assess the Pt coordination sphere of 1/SiO_2_–Al_2_O_3_ and 1/SBA-15. Due to the low concentration of the phosphine compounds on the surface, DNP was used to boost the sensitivity of the ^31^P{^195^Pt} *J*-resolved NMR experiments. Fig. 7A shows ^1^H spin echo NMR spectra of 1/SiO_2_–Al_2_O_3_ and 1/SBA-15 recorded with a 25 kHz MAS frequency. Both materials show negatively shifted ^1^H NMR signals attributed to hydride protons. The chemical shifts of the hydride peaks are −35 ppm for 1/SiO_2_–Al_2_O_3_ and –26 ppm for 1/SBA-15. The hydride ^1^H NMR signals also show splittings attributable to ^1^H–^195^Pt spin–spin couplings (2.6 and 1.7 kHz for 1/SiO_2_–Al_2_O_3_ and 1/SBA-15, respectively). For 1/SiO_2_–Al_2_O_3_ we confirmed that the satellite peaks dephase in a ^1^H{^195^Pt} *J*-resolved experiment (Fig. S5†), which confirms the presence of platinum hydrides in these supported compounds. A ^1^H{^195^Pt} *J*-resolved experiment was not conducted for 1/SBA-15 due to the poorer ^1^H signal-to-noise.

We performed control room temperature ^31^P ssNMR experiments on samples of 1/SBA-15 and 1/SiO_2_–Al_2_O_3_ impregnated with TEMPO solutions (Fig. S6–S7†). We observed decreased ^31^P NMR sensitivity but no degradation towards C–H activated or other complexes. Therefore, the degradation to C–H activated species likely occurred while the samples were held under vacuum in sealed storage tubes for the weeks it took to ship the samples from Germany to the USA to perform DNP experiments. Exposure to the biradical does appear to cause some degradation to phosphine oxide and protonated phosphine decomposition products (*vide infra*). However, under the conditions needed for DNP enough of the Pt–H species are present for to measure the ^195^Pt ssNMR spectra.

Fig. 7B shows DNP-enhanced ^31^P{^195^Pt} *J*-resolved control (*S*_0_), dephasing (*S*), and difference (*S*_0_ − *S*) spectra. The ^31^P NMR spectrum of 1/SiO_2_–Al_2_O_3_ shows the same peak at 82 ppm due to the [HPt(P^*t*^Bu_3_)_2_] fragment, along with new peaks at 71 ppm (OP^*t*^Bu_3_) and 57 ppm (HP(^*t*^Bu)_3_^+^).^65,73^ These new peaks were due to partial decomposition from the DNP biradical solution (Fig. S7–S8†). The ^31^P NMR of 1/SBA-15 shows the expected signal of the [HPt(P^*t*^Bu_3_)_2_] fragment at 72 ppm along with new signals at 52, 25, and 4 ppm. The ^31^P–^195^Pt *J*-couplings of both of the Pt–H species were measured to be 2433 and 2908 Hz for 1/SiO_2_–Al_2_O_3_ and 1/SBA-15, respectively, which are close to the expected value of *ca.* 2500–3000 Hz for a ^31^P–^195^Pt(II) bond. The ^31^P NMR signals at 52 and 25 ppm (with close to 1 : 1 ratio) can be assigned as the C–H activated [*κ*^2-*t*^Bu_2_P(CMe_2_CH_2_–)Pt(P^*t*^Bu_3_)]^+^ complex based on the similarity to NMR spectra of known cyclometallated Pt complexes.^72^ While the exact nature of the decomposition peak at 4 ppm on 1/SBA-15 is at this point unclear, this is consistent with higher oxidation of the phosphine (*e.g.* to phosphites or phosphonates) upon reaction with TEKPol.^74^ Peak fits of the ^1^H–^31^P CPMAS spectra for 1/SiO_2_–Al_2_O_3_ and 1/SBA-15 are shown in Fig. S9 and Table S1.† These fits demonstrate that while the Pt–H is still the most prominent species in 1/SiO_2_–Al_2_O_3_ (*ca.* 60%), the decomposition of 1/SBA-15 is significantly greater, showing that the proportion of Pt–H to C–H activated species are both *ca.* 30% of the total phosphorus.^72^

Lastly, we performed variable ^195^Pt offset ^31^P{^195^Pt} *J*-resolved experiments to measure the ^195^Pt CS tensor. These experiments were challenging for a number of reasons. First, the ^195^Pt spinning sidebands are likely broadened by several kilohertz, meaning that at the MAS frequency of 12.5 kHz achievable in the DNP setup the sidebands will likely be overlapped with one another. Consequently, the dephasing profiles will roughly trace out the MAS spectrum sideband pattern, but it is not possible to measure sideband positions or isotropic ^195^Pt chemical shifts with high precision. Second, we also had to use ^195^Pt saturation pulses with durations of 120 μs and 35 kHz RF fields to induce enough dephasing in the ^31^P{^195^Pt} *J*-resolved experiments (Fig. S10†). The use of these pulse conditions causes distortions of the dephasing profile, making it challenging to accurately determine *δ*_22_ or *κ*. Finally, under DNP conditions, the ^31^P signals suffered from inhomogeneous broadening on the order of a few kHz, reducing NMR sensitivity. To overcome the inhomogeneous broadening, a ^31^P CPMG echo train detection was used in the ^31^P{^195^Pt} *J*-resolved experiment (Fig. S11†).

Fig. 7D shows the ^195^Pt NMR spectra reconstructed from the ^31^P{^195^Pt} *J*-resolved experiments for both 1/SiO_2_–Al_2_O_3_ and 1/SBA-15 along with SIMPSON-simulated fits. For the 1/SiO_2_–Al_2_O_3_, where the Pt–H is the primary surface species, the ^195^Pt NMR signature matches closely with complexes 2–4 (*δ*_iso_ = −4841 ppm, *Ω* = 5680 ppm, *κ* = −0.6). The spectrum of 1/SBA-15 is somewhat more complicated and had to be fit to two Pt sites, one with a small CSA (site 1: *δ*_iso_ = −4441 ppm, *Ω* = 4080 ppm, *κ* = 0.0) and the other with a large CSA (site 2: *δ*_iso_ = −3541 ppm, *Ω* = 7980 ppm, *κ* = 0.0). We note that for both site 1 and site 2, there is large uncertainty in the fit of *κ*, so it is set equal to 0.0 (Fig. S12†). The spectrum of site 1 was obtained by monitoring the dephasing of the spikelets in the ^31^P CPMG spectrum centered around 90 ppm (mainly from Pt–H surface complexes) while the spectrum of site 2 is from the lower frequency spikelets around 50 ppm (mainly from C–H activated complex). Hence, we believe that site 1 is the Pt hydride and site 2 is the C–H activated species (Fig. S9 and S11†). Dephasing of the ^31^P resonance from the cyclometallated phosphine at 25 ppm was not observed due to a lower *J*-coupling constant (*J*_Pt–P_ ≈ 2000 Hz) in comparison to Pt–H complexes (*J*_Pt–P_ ≈ 3000 Hz).

### DFT modeling of surface-supported complexes

**Fig. 8:**
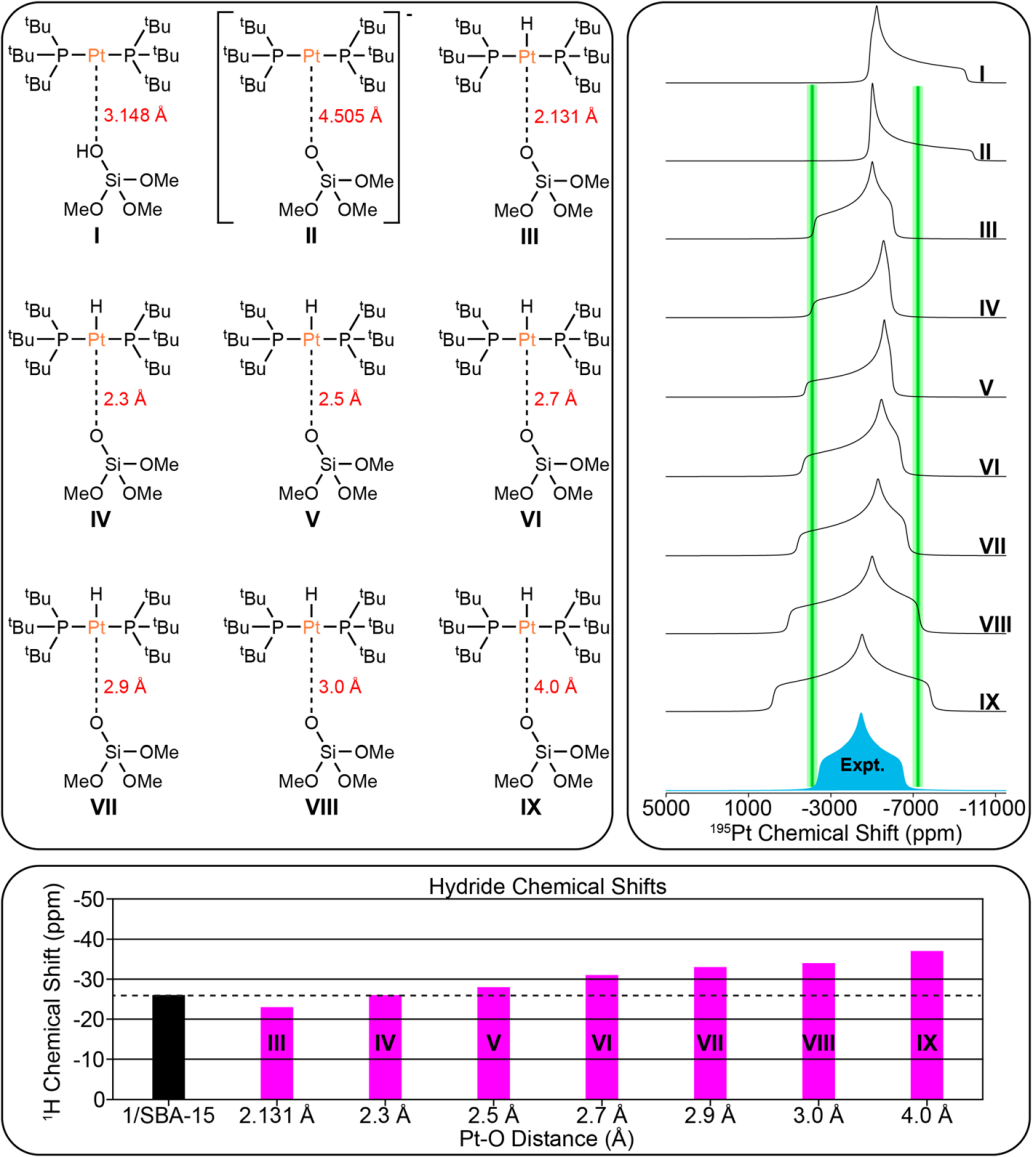
(Top left) Possible structural models of 1/SBA-15 used for DFT calculations. The Pt–O bond lengths are indicated. (Top right) Comparison of DFT calculated ^195^Pt static lineshapes for each model and experimental ^195^Pt static lineshape for 1/SBA-15. Green bars that are 600 ppm wide are shown at *δ*_11_ and *δ*_33_ to show the approximate combined error based upon fits of experimental dephasing profiles and standard deviation of DFT calculations. Green bars are not shown for *δ*_22_ due to the large uncertainty in the skew (*κ*). (Lower panel) Comparison of experimental (black) and calculated hydride ^1^H chemical shifts (pink).

**Fig. 9:**
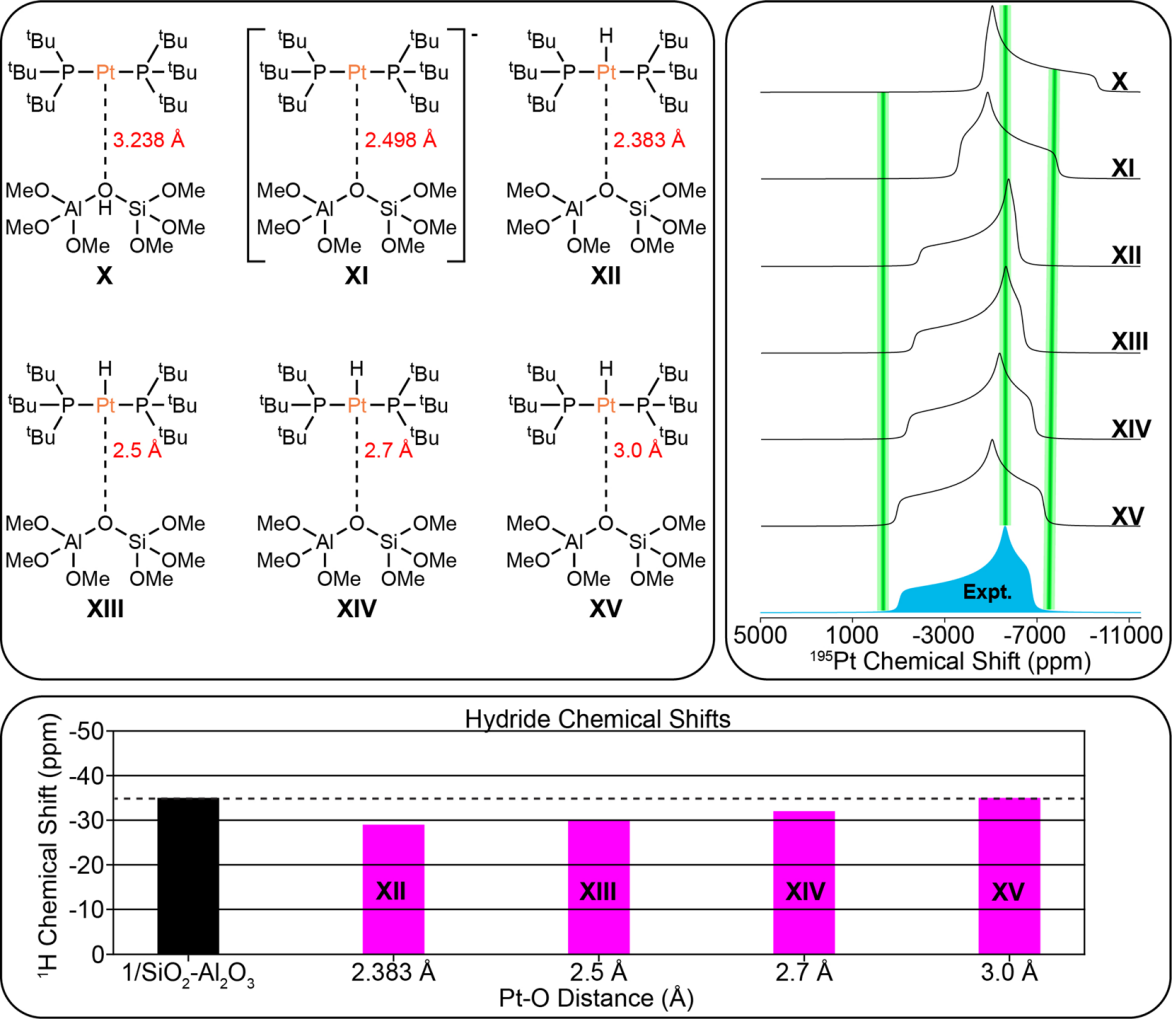
(Top left) Possible structural models of 1/SiO_2_–Al_2_O_3_ used for DFT calculations. The Pt–O bond lengths are indicated. (Top right) Comparison of DFT calculated ^195^Pt static lineshapes for each model and experimental ^195^Pt static lineshape for 1/SiO_2_–Al_2_O_3_. Green bars that are 600 ppm wide are shown at *δ*_11_, *δ*_22_, and *δ*_33_ to show the approximate combined error based upon fits of experimental dephasing profiles and standard deviation of DFT calculations. (Lower panel) Comparison of experimental (black) and calculated hydride ^1^H chemical shifts (pink).

To understand the nature of the Pt–O bonds of the surface supported platinum hydrides, we created nine DFT structural models for 1/SBA-15 (Fig. 8) and six for 1/SiO_2_–Al_2_O_3_ (Fig. 9) with varying Pt–O bond lengths. For these calculations, [HPt(P^*t*^Bu_3_)_2_]^+^ fragments were ligated by [(MeO)_3_Al–O–Si(OMe)_3_]^−^ or [OSi(OMe)_3_]^−^ (structural analogs of the SiO_2_–Al_2_O_3_ and SBA-15 surface oxygen atoms, respectively) and were geometrically optimized by DFT, then the NMR parameters were calculated. However, these model ligands do not have the same steric and electronic behavior as the true surface sites. Therefore, we subsequently geometrically optimized the same structure with constrained Pt–O bond lengths and calculated their NMR parameters. The Pt–O bond length was successively increased from the DFT calculated equilibrium distances for each model ligand in order to see the effect of lengthening the Pt–O bond on the NMR spectra and more accurately predict the true Pt–O bond length. We also carried out the same calculations for Pt(0) complexes coordinating to either surface OH or anionic surface sites using the same surface model ligands. Table S2† contains a detailed list of the DFT-calculated hydride ^1^H and ^31^P chemical shifts, and the ^1^H–^195^Pt and ^31^P–^195^Pt *J*-couplings for all structural models. The ^31^P chemical shifts and ^31^P–^195^Pt *J*-couplings will not be discussed further because they change little between the structural models. The ^1^H–^195^Pt *J*-couplings differ by several hundred Hertz for each model, but due to the poor signal-to-noise in the room temperature ^1^H spin echo spectra (Fig. 7A), it was difficult to accurately measure the ^1^H–^195^Pt *J*-couplings, so they will not be discussed either. Based on our calculations, the ^195^Pt CS tensors and ^1^H chemical shift of the hydride provide crucial structural information about the surface supported complexes.

Fig. 8 shows the comparison of the experimental ^195^Pt static lineshape to the calculated lineshapes of the model structures as well as the calculated hydride chemical shifts for 1/SBA-15. We note that there is large uncertainty in the least-squares fit of *κ*/*δ*_22_ for 1/SBA-15, so it is not considered here. The calculations predict that the ^195^Pt CSA increases as the Pt–O distance increases past the calculated equilibrium distance of 2.131 Å. This prediction is consistent with our experiments that showed the three-coordinate complex 3 has a much larger CSA than either of the four-coordinate complexes 2 or 4. The hydride shift also becomes more negative as the Pt–O distance increases (−23 ppm for model III and −37 ppm for model IX). The least squares fit of the ^195^Pt CSA indicates that the calculated ^195^Pt lineshape of model III matches most closely with the experimental spectrum, while the hydride ^1^H chemical shift matches best with model IV. Therefore, we conclude the surface supported Pt–H complex present in 1/SBA-15 most likely has Pt–O distances between 2.1 Å and 2.3 Å.

The DFT structural models for 1/SiO_2_–Al_2_O_3_ are shown in Fig. 9. DFT calculations predicted an equilibrium Pt–O bond length of 2.383 Å. The comparison of the experimental ^195^Pt static lineshape to the calculated lineshapes of the model structures as well as the calculated hydride chemical shifts are shown. DFT calculations predict similar trends between the Pt–O bond length and the ^195^Pt CSA and hydride ^1^H chemical shift as was seen above for 1/SBA-15. The least squares fit of the ^195^Pt CSA indicates that the ^195^Pt lineshape of model XIV matches best with the experimental spectrum, and the hydride chemical shift of model XV matches with the experimental chemical shift. We therefore conclude that the Pt–O distance is likely between 2.7 and 3.0 Å for 1/SiO_2_–Al_2_O_3_.

Our experimental observations and DFT calculations demonstrate a few key differences between the Pt–H complexes on the different supports. Previous NMR and FTIR studies suggest that *ca.* 90% of surface Si–OH groups on SiO_2_–Al_2_O_3_ neighbor nearby Al lewis acid sites, explaining the enhanced acidity of SiO_2_–Al_2_O_3_ as compared to SiO_2_.^75–79^ This higher acidity of leads to a higher Pt loading per unit surface area on SiO_2_–Al_2_O_3_ by making the thermodynamics of the O–H oxidative addition to Pt more favorable. Consequently, the Pt loadings on SBA-15 and SiO_2_–Al_2_O_3_ are similar, despite SBA-15 having a much larger surface area than SiO_2_–Al_2_O_3_. Consistent with this finding, the IR spectra of SiO_2_–Al_2_O_3_ before and after immobilization of 1 shows a decrease in the intensity of *ν*_OH_ by about half (Fig. S16†). In contrast, the intensity of *ν*_OH_ on SBA-15 shows a smaller intensity change after immobilization of 1, suggesting that only a small percentage of isolated Si–OH groups react with 1 (Fig. S17†). In addition, we expect that the increased acidity of the OH groups on SiO_2_–Al_2_O_3_ leads to longer Pt–O bond distances than for 1/SBA-15. This is because the higher acidity of the [Al–OH–Si] groups makes [Al–O–Si]^−^ more weakly coordinating than [Si–O]^−^, leading to longer Pt–O bond lengths on SiO_2_–Al_2_O_3_. Longer Pt–O distances on SiO_2_–Al_2_O_3_ also correlate with the propensity for the complexes to undergo C–H activation of the ^*t*^Bu groups, which is dependent on the *trans*-influence of the X-type ligand (*vide supra*).^11^ This fact explains why 1/SiO_2_–Al_2_O_3_ shows no C–H activation while 1/SBA-15 does undergo cyclometallation (Scheme 1).

We previously measured a Pt–O distance of 2.01(3) Å *via* Pt L3 edge EXAFS of 1/SiO_2_–Al_2_O_3_, which is seemingly at odds with our results here.^13^ However, given that the complex undergoes C–H activation, it may be that the 2.01(3) Å bond distance actually corresponded to the Pt–C bond of the cyclometallated complex, which formed either during shipment of the sample under high vacuum or (more likely) in the X-ray beam of the XAS measurements. This explanation is supported by the experimental Pt–C bond length of *ca.* 2.06 Å of a similar cyclometallated Pt(P^*t*^Bu_3_) complex.^72^ This result highlights the need for complementary characterization strategies such as NMR spectroscopy and EXAFS in order to arrive at a clear understanding of surface structure.

## Conclusions

In conclusion, this study demonstrates that sideband selective experiments with selective long pulses can be used for the rapid reconstruction of ^195^Pt ssNMR spectra of supported platinum hydrides on different metal oxides (SiO_2_ and SiO_2_/Al_2_O, ∼2 Pt wt% loading). With ^1^H{^195^Pt} and ^1^H–^31^P{^195^Pt} *J*-resolved and *J*-HMQC experiments it was possible to rapidly measure ^195^Pt ssNMR spectra of the molecular complexes 1–4. The ^1^H, ^31^P and ^195^Pt NMR fingerprints provide direct insight into the oxidation state and coordination environment of the molecular complexes. With this information it was possible to probe the structure of surface species formed by supporting 1 on SBA-15 or SiO_2_–Al_2_O_3_.

**Fig. 10:**
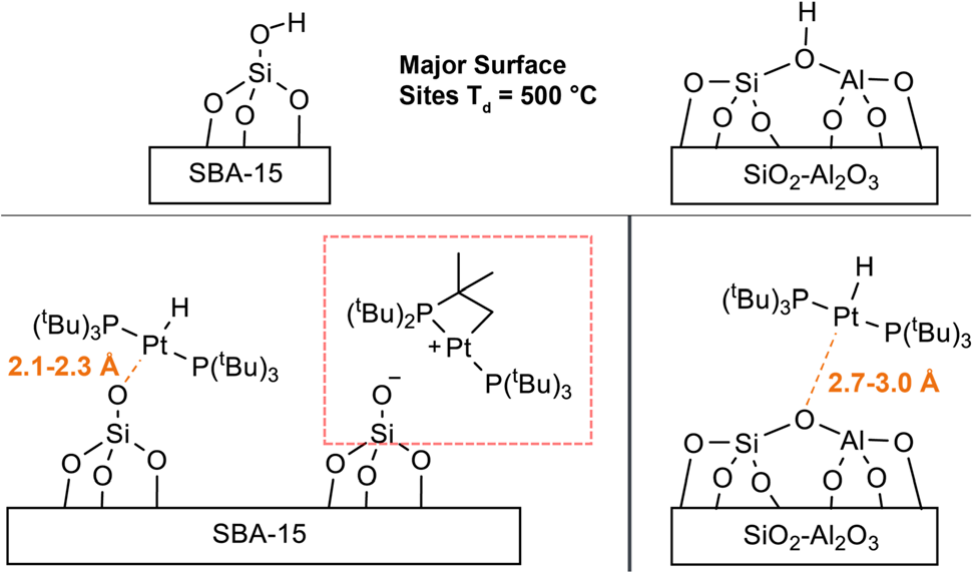
Summary of structural information for supported compounds. Major surface sites after dehydroxylation at 500 °C on the respective metal oxide before reaction with 1. When 1 is supported on SBA-15 two different Pt surface species are formed: a bisphosphine hydride and a CH-activated phosphine complex. Calculations of the ^195^Pt CSA suggest that the Pt–O bond length is between 2.1 to 2.3 Å for the bisphosphine hydride. When 1 is supported on SiO_2_–Al_2_O_3_ a bisphosphine hydride is the major surface species. The Pt–O bond length is between 2.7 and 3.0 Å.

Monitoring the ^31^P signal dephasing as a function of ^195^Pt offset in *J*-resolved experiments allowed for the determination of the ^195^Pt CS tensor of the surface Pt species in 1/SiO_2_–Al_2_O_3_ and 1/SBA-15. Numerical fits of ^1^H–^31^P{^195^Pt} *J*-resolved curves also allowed the accurate determination of ^31^P–^195^Pt *J*-coupling constants in the supported complexes (2433 Hz and 2908 Hz for 1/SiO_2_–Al_2_O_3_ and 1/SBA-15, respectively). These values align closely with the expected value of approximately 2500 Hz for a ^31^P–^195^Pt(II) bond. Comparing experimental and DFT calculated NMR parameters suggested that the bond distance between Pt and O for 1/SiO_2_–Al_2_O_3_ was about 2.7 Å. This distance does not correspond to that of a typical covalent bond, which is around 2 Å, indicating the presence of a 3-coordinated complex on the surface of 1/SiO_2_–Al_2_O_3_ (Fig. 10). Several additional data, including the hydride ^1^H NMR signal at −36 ppm, the ^195^Pt CSA, and the ^31^P–^195^Pt *J*-coupling constants, show the characteristic features of a 3-coordinate complex. Room temperature ^31^P ssNMR spectra indicate the presence of two surface species on 1/SBA-15, one of which is a 4-coordinated bisphosphine hydride complex with a Pt–O bond length between 2.1 Å to 2.3 Å and a proton shift of −27 ppm (Fig. 10). The other species is a decomposition product, which was assigned to a cyclometallated compound, formed by C–H activation. The use of sideband selective ^195^Pt ssNMR experiments allowed the differentiation of two platinum species.

Overall, the combination of experimental and computational techniques presented in this paper offers a comprehensive characterization of these surface-supported Pt complexes. The knowledge gained from this study contributes to the understanding of the coordination geometry, Pt oxidation states, and electronic environments of these complexes. This knowledge can guide the design and optimization of heterogeneous catalysts based on supported Pt species, facilitating the development of efficient catalytic systems for various chemical transformations.

## Experimental section

### General

All air- and moisture-sensitive materials were manipulated under an atmosphere of oxygen-free dry argon using a MBraun glovebox and standard schlenkline techniques.^80^ Spectrograde acetone was stored over molecular sieve (3 A) 24 h prior to use. Dichloromethane and pentane were used directly form the SPS and dry benzene was used as bought from Sigma Aldrich (anhydrous benzene, 99.99%). *Materials*: bis(tri-*tert*-butyl-phosphine)platinum(0) 1 and the platinum hydrides 2–4 were synthesized according to an adapted literature procedures.^64^

### Preparation of complex 2: *trans*-PtHCl[P(^*t*^Bu)_3_]_2_

To a stirred solution of Pt(0)(P^*t*^Bu_3_)_2_1 (650 mg, 1.1 mmol) in toluene, HCl dissolved in toluene (2 eq., 0.2 mmL, 12 M) was added slowly. It was stirred for 20 minutes and dried under vacuum and crystallized from *n*-pentane to give a white powder (570 mg, 0.9 mmol, 83%). ^1^H NMR (400 MHz, benzene-*d*_6_): *δ* = 1.58–1.51 (m, 54H), −19.20 (tt, *J*_H–Pt_ = 1072 Hz, *J*_H–P_ 12.9 Hz, ^1^H) ppm. ^31^P NMR (162 MHz, benzene-*d*_6_): *δ* = 75.69 (t, *J*_P–Pt_ = 2951 Hz).

### Preparation of complex 3: *trans*-PtH[P(^*t*^Bu)_3_]_2_ BF_4_

Complex 2 (450 mg, 0.7 mmol) was dissolved in dry acetone (3 mL) and AgBF_4_ (137.70 mg, 0.7 mmol) dissolved in acetone (2 mL) was added slowly. The precipitate AgCl was filtered off and the filtrate was dried under vacuum. After precipitation from CH_2_Cl_2_/*n*-pentane a bright yellow powder was obtained (230 mg, 0.33 mmol, 50%). It was crystalized from CH_2_Cl_2_/*n*-pentane. ^1^H NMR (400 MHz, chloroform-*d*) *δ* 1.29–1.15 (m, 54H), −35.63 (t, *J*_H–Pt_ = 2638 Hz, ^1^H) ppm. ^31^P NMR (162 MHz, chloroform-*d*): *δ* = 85.58 ppm (t, *J*_P–Pt_ = 2625) ppm.

### Preparation of complex 4: *trans*-PtH[CH_3_CN][P(^*t*^Bu)_3_]_2_ BF_4_

3 was dissolved in CH_2_Cl_2_ and an excess of CH_3_CN (>1 eq.) was added. The solution was dried under vacuum and a white powder was yield quantitatively. It was crystalized from CH_2_Cl_2_/*n*-pentane. ^1^H NMR (400 MHz, CDCl_3_): *δ* = 1.48–1.37 (m, 54H), −19.20 (t, *J*_H–Pt_ = 1049 Hz, *J*_H–P_ = 12.4 Hz, 1H) ppm. ^31^P NMR (162 MHz, chloroform-*d*): *δ* = 80.07 (t, *J*_P–Pt_ = 2810) ppm.

### Preparation of 1/SBA-15

The reaction of 1 with SBA-15 was done using the double-Schlenk technique. A solution of 1 (30 mg, 0.058 mmol) in toluene (3 mL) was frozen in one finger of the double Schlenk. The other finger was filled with SBA-15 (dehydroxylated at 500 °C, 500 mg). The Schlenk was evacuated and under static vacuum the solution was unfrozen and added to the powder. The reaction was stirred for 20 h at room temperature. The mixture was filtered and washed with *n*-pentane (3 × 5 mL) before dried under high vacuum (10^−5^ mbar). An off-white powder was obtained (loading: 0.1 mmol g^−1^, determined by ICP-OES). Following synthesis, room temperature MAS ^1^H ssNMR spectra were acquired with an MAS frequency of 7 kHz. The ^1^H ssNMR spectrum showed a diagnostic hydride ^1^H NMR signal at −26.6 ppm and a ^1^H-^195^Pt *J*-coupling of 1034 Hz.

### Preparation of 1/SiO_2_–Al_2_O_3_

A solution of 1 (30 mg, 0.058 mmol) in pentane (1 mL) was added to suspension of SiO_2_–Al_2_O_3_ (dehydroxylated at 500 °C) in pentane (2 mL). After 20 h stirring at room temperature the mixture was filtered and washed with *n*-pentane (3 × 5 mL) before drying under high vacuum (10^−5^ mbar). An off-white powder was obtained (loading: 0.1 mmol g^−1^, determined by ICP-OES). Following synthesis, a room temperature ^1^H MAS ssNMR spectrum was acquired with a 10 kHz MAS frequency. The 1H ssNMR spectrum showed a diagnostic hydride ^1^H NMR signal at −36.3 ppm and a ^1^H-^195^Pt *J*-coupling of 2510 Hz.

### Room temperature solid-state NMR experiments

All experiments were performed with a 9.4 T wide bore magnet equipped with a Bruker Avance III HD console. Chemical shifts were indirectly referenced to adamantane using the ^1^H shift at 1.82 with respect to TMS in CDCl_3_ ppm using the IUPAC-recommended chemical shift convention.^81^ The probe was not retuned for any sideband selective experiment. The ^195^Pt RF field was calibrated using the Bloch–Siegert shift method.^82^ The RF field strength of the ^195^Pt saturation pulses for the sideband selective experiments varied depending on the MAS frequency and the saturation pulse length that was used. Numerical simulations suggest that the optimal RF fields are within a few kilohertz of those that were used experimentally (Fig. S18 and Table S6†).

### DNP-SENS solid-state NMR experiments

All experiments were performed with a wide bore 9.4 T/263 GHz DNP spectrometer^83^ equipped with a Bruker Avance III HD console. Chemical shifts were indirectly referenced to the ^13^C chemical shift of tetrakis(trimethylsilyl)silane at 2.75 ppm (with respect to TMS (*δ*(^1^H) = 0 ppm) in 98% CDCl_3_). The ^195^Pt RF field was calibrated by scaling the ^13^C π/2 pulse length, which was calibrated directly on tetrakis(trimethylsilyl)silane by nutating the pulse length.

### Room temperature ^1^H{^195^Pt} experiments

Complex 3 was packed into a 1.3 mm zirconia rotor in a glovebox then spun with nitrogen gas. A 1.3 mm Bruker HX probe and a MAS frequency of 50 kHz was used. Before experiments began, the magic angle was precisely calibrated by minimizing the splitting in the ^2^H NMR spectrum of deuterated oxalic acid.^84^^1^H 90 and 180° pulse used durations of 2.5 and 5 μs, respectively. A ^1^H{^195^Pt}2D *J*-HMQC with a rotor-synchronized *t*_1_-evolution period and hyper-complex states-TPPI acquisition^85^ was done to determine the exact frequency of a ^195^Pt spinning sideband so that sideband selective experiments could be performed. The sideband selective experiments were performed with 32 scans, 1.55 s recycle delay (1.3 × *T*_1_), 0.4 ms *J*-evolution period (^1^*J*(^1^H–^195^Pt) = ∼2500 Hz), and 31 sub-spectra. 60 μs SL pulses with an 8 kHz and 6 kHz RF field were employed for *J*-resolved and *J*-HMQC, respectively.

### Room temperature ^1^H–^31^P{^195^Pt} experiments on 1

Complex 1 was packed into a 2.5 mm zirconia rotor in a glovebox then spun with nitrogen gas. A Bruker 2.5 mm HXY probe configured in ^1^H–^31^P–^195^Pt mode and a MAS frequency of 25 kHz were used. Before experiments began, the magic angle was precisely calibrated by maximizing the intensity of the second spinning sideband of the ^79^Br spectrum of KBr.^86^^1^H 90 and 180° pulse used durations of 2.5 and 5 μs, respectively. The ^1^H → ^31^P CP contact time was set to 2.5 ms and optimal ^1^H and ^31^P spinlock RF powers of 105 and 70 kHz, respectively, were used; the ^1^H RF power was ramped from 90 to 100%. A ^1^H–^31^P{^195^Pt} 2D *J*-HMQC experiment with a rotor-synchronized *t*_1_-evolution period and hyper-complex states-TPPI acquisition^85^ was used to determine the exact frequency of a ^195^Pt spinning sideband so that sideband selective experiments could be performed. Sideband selective experiments used 80 μs SL ^195^Pt pulses with a 6 kHz RF field or 5 kHz RF field for *J*-resolved and *J*-HMQC, respectively. 128 scans and 512 scans were acquired for *J*-resolved and *J*-HMQC, respectively The recycle delay was 0.66 s (1.3 × *T*_1_), the *J*-evolution period was 0.22 ms (^1^*J*(^31^P–^195^Pt) = 4400 Hz), and 31 sub-spectra were acquired. 100 kHz ^1^H SPINAL-64 decoupling was applied during the *J*-evolution periods and signal acquisition.^87^ The same ^31^P CP conditions and ^1^H decoupling conditions were used for room temperature ssNMR experiments on compound **2**–**4**.

### Room temperature ^1^H–^31^P{^195^Pt} experiments on 2

Sideband selective experiments used 120 μs SL pulses with 7 kHz and 4 kHz RF fields for *J*-resolved and *J*-HMQC, respectively. The recycle delay was 1.17 s (1.3 × *T*_1_), the *J*-evolution period was 0.22 ms (^1^*J*(^31^P–^195^Pt) = ∼3000 Hz), and 56 sub-spectra were acquired. 32 scans were acquired at each ^195^Pt offset.

### Room temperature ^1^H{^195^Pt} experiments on 3

Sideband selective experiments used 60 μs SL pulses with 8 kHz and 6 kHz RF fields for *J*-resolved and *J*-HMQC, respectively. The recycle delay was 1.55 s (1.3 × *T*_1_), the *J*-evolution period was 0.4 ms (^1^*J*(^31^P–^195^Pt) = ∼2500 Hz), and 31 sub-spectra were acquired. 32 scans were acquired at each ^195^Pt offset.

### Room temperature ^1^H–^31^P{^195^Pt} experiments on 4

Sideband selective experiments used 80 μs SL pulses with 7 kHz and 5 kHz RF for *J*-resolved and *J*-HMQC, respectively. The recycle delay was 1.43 s (1.3 × *T*_1_), the *J*-evolution period was 0.16 ms (^1^*J*(^31^P–^195^Pt) = ∼6250 Hz), which was not optimal, and 66 sub-spectra were acquired. 64 scans were acquired at each ^195^Pt offset. A CPMG echo train was added at the end of the sideband selective pulse sequences for detection to increase sensitivity.

### DNP-SENS ^1^H–^31^P{^195^Pt} experiments on 1/SiO_2_–Al_2_O_3_ and 1/SBA-15

The DNP samples of 1/SiO_2_–Al_2_O_3_ and 1/SBA-15 were prepared in a glovebox and impregnated with a solution of 10 mM TEKPol^88^ in 1,1,2,2 tetrachloroethane (TCE) *via* the incipient wetness method and packed into a 3.2 mm sapphire rotor. The rotor was then inserted into a low-temperature Bruker 3.2 mm HXY MAS DNP probe configured in ^1^H–^31^P–^195^Pt mode which was precooled to 100 K, then spun with an MAS frequency of 12.5 kHz. The ^1^H → ^31^P CP contact time was set to 2 ms and optimal ^1^H and ^31^P spinlock RF powers of 40 and 72 kHz, respectively, were used; the ^1^H RF power was ramped from 90 to 100%. ^1^H–^31^P{^195^Pt} *J*-resolved experiments to measure ^31^P–^195^Pt *J*-couplings were performed with a WURST-80 (ref. 35) saturation pulse on ^195^Pt. The WURST-80 pulse was 25 μs duration, used a RF field of *ca.* 120 kHz, and swept over a total frequency range of 1.25 MHz. The WURST pulse was found to give superior dephasing as compared to a rectangular pulse. 100 kHz ^1^H SPINAL-64 decoupling was applied during the *J*-evolution periods and signal acquisition.^87^ For ^195^Pt variable offset ^1^H–^31^P{^195^Pt} *J*-resolved experiments rectangular ^195^Pt saturation pulses that were 120 μs (1.5 × *τ*_r_) in duration were used with an RF field of 35 kHz. The ^195^Pt saturation pulse offset was incremented in steps of 25 kHz while retuning the ^195^Pt channel on the probe every 250 kHz; 69 sub-spectra were acquired. The total echo duration was set to 0.32 ms which is approximately the inverse of the ^31^P–^195^Pt *J*-coupling. For all experiments, the recycle delay was 2.21 s for 1/SiO_2_–Al_2_O_3_ and 4.43 s for 1/SBA-15. 128 scans were acquired at each ^195^Pt offset.

### Room temperature ssNMR experiments on 1/SiO_2_–Al_2_O_3_ and 1/SBA-15

1/SiO_2_–Al_2_O_3_ and 1/SBA-15 were packed into 2.5 mm zirconia rotors inside of a glovebox and then spun with nitrogen gas. A Bruker 2.5 mm HX probe configured in double mode was used. The ^1^H spin echo (π/2–τ–π–τ) spectra were acquired with a (1.3 × *T*_1_) recycle delay (2 s for 1/SiO_2_–Al_2_O_3_ and 1.42 s for 1/SBA-15). The ^1^H spectra were acquired with 4096 and 16 384 scans for 1/SiO_2_–Al_2_O_3_ and 1/SBA-15, respectively.

### Numerical SIMPSON simulations

Numerical simulations were performed using SIMPSON v4.2.1.^89–91^ The SIMPSON input codes are provided with the archived data. All pulses in the files were of finite duration, except the ^1^H and ^31^P π/2 pulses, which were ideal. The rep320 crystal file was used for all simulations. The number of gamma angles was set to 13. The heat maps in the ESI† were constructed using MATLAB_R2021B. The 1D spectra were processed with Python using Microsoft Visual Studio Code as the interpreter.

### DFT calculations

For complexes 1–4, hydrogen atom coordinates were geometrically optimized in CASTEP^54^ using the PBE-GGA functional,^92^ TS dispersion correction scheme,^93^ and ultra-soft pseudopotentials.^94,95^ The coordinates for the models of 1/SiO_2_–Al_2_O_3_ and 1/SBA-15 were geometrically optimized with AMS 2021. All NMR calculations were performed using AMS 2021 with the hybrid PBE0 functional^56,57^ and a Slater-type basis set of triple-ζ with two polarization functions.^55^ Relativistic effects were treated by zero order regular approximation (ZORA)^96–99^ with spin–orbit relativity level.

## Data availability

The data that support the findings of this study are openly available at https://doi.org/10.5281/zenodo.12773732 or https://zenodo.org/records/12773732. Data includes experimental NMR datasets and pulse programs (TopSpin format), SIMPSON simulation files and AMS 2021 files. Solution NMR spectra of molecular compounds, 1D ^1^H and ^31^P MAS NMR spectra, crystallographic data and infrared spectra are available at https://doi.org/10.18419/darus-4580.

## Author contributions

B. A. A. performed solid-state NMR experiments, performed DFT calculations and analyzed data. E. J. W. synthesized compounds, characterized them by standard techniques and assisted with solid-state NMR experiments. S. K. and J. K. performed DFT calculations. W. F. obtained single-crystal X-ray structures of compounds. A. J. R. and D. P. E. directed and oversaw all research. The manuscript was written through the contributions of all authors.

## Conflicts of interest

There are no conflicts of interest to declare.

## Supplementary Material

SC-016-D4SC06450J-s001

SC-016-D4SC06450J-s002
