# Supplementary material for: Structural characterization of surface immobilized platinum hydrides by sensitivity-enhanced 195Pt solid state NMR spectroscopy and DFT calculations
Source: Chem Sci. 2024 Nov 20;16(3):1271–87. doi: 10.1039/d4sc06450j (PMC11638849; doi:10.1039/d4sc06450j)
Supplement: SC-016-D4SC06450J-s001 [file SC-016-D4SC06450J-s001.pdf]

Supplementary Information (SI) for Chemical Science.
This journal is © The Royal Society of Chemistry 2024

Supporting Information for:

Structural Characterization of Surface Immobilized Platinum Hydrides by Sensitivity-Enhanced ^{195}Pt Solid State NMR Spectroscopy and DFT Calculations

Benjamin A. Atterberry, [1, 2†] *Erik J. Wimmer,*[3†] *Sina Klostermann,*[3] *Wolfgang Frey,*[3] *Johannes Kästner,*[3] *Deven P. Estes,*[3*] *and Aaron J. Rossini* [1,2*]

†These authors contributed equally.

[1]*Iowa State University, Department of Chemistry, Ames, IA, USA, 50011*
[2]*US DOE Ames National Laboratory, Ames, Iowa, USA, 50011*
[3]*University of Stuttgart, Department of Chemistry, Stuttgart, Baden-Württemberg, Germany, 70569*

Corresponding Authors:
*Emails: arossini@iastate.edu, deven.estes@itc.uni-stuttgart.de

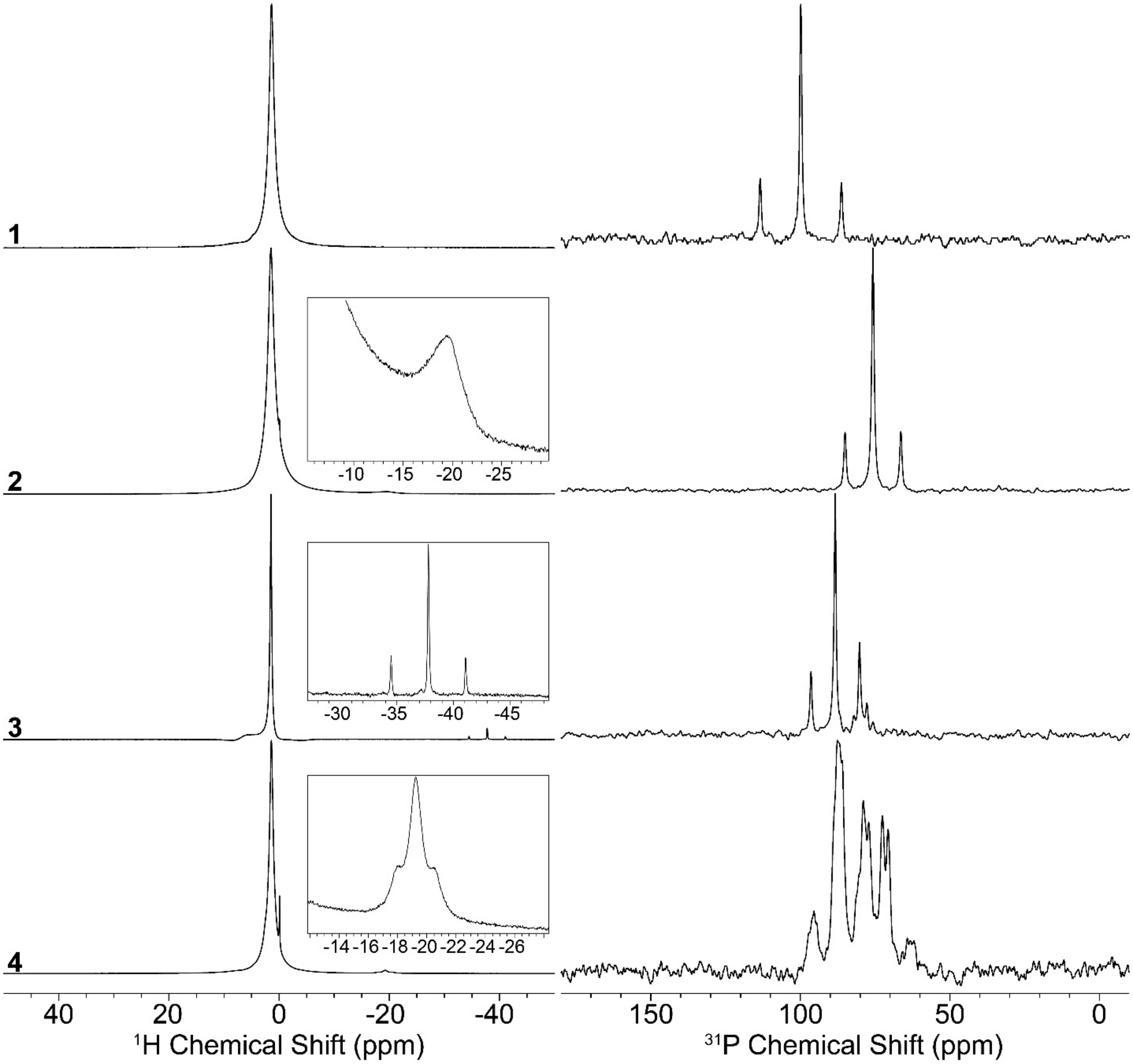

Figure S1: ^{1}H spin echo and ^{1}H-^{31}P CPMAS spectra for molecular compounds **1** through **4**.

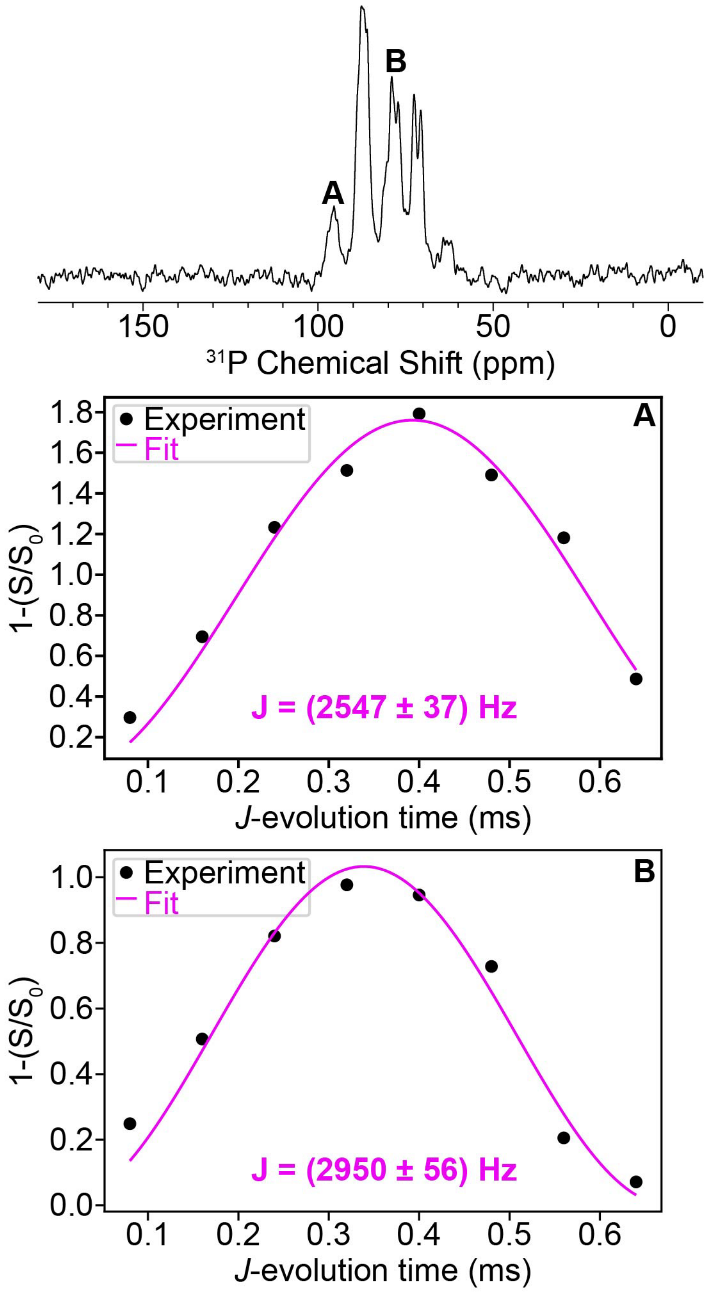

Figure S2: ^{31}P{^{195}Pt} *J*-evolution plots of **4** for different ^{31}P peaks. **A** and **B** denote the 95 ppm and 78 ppm peaks, respectively.

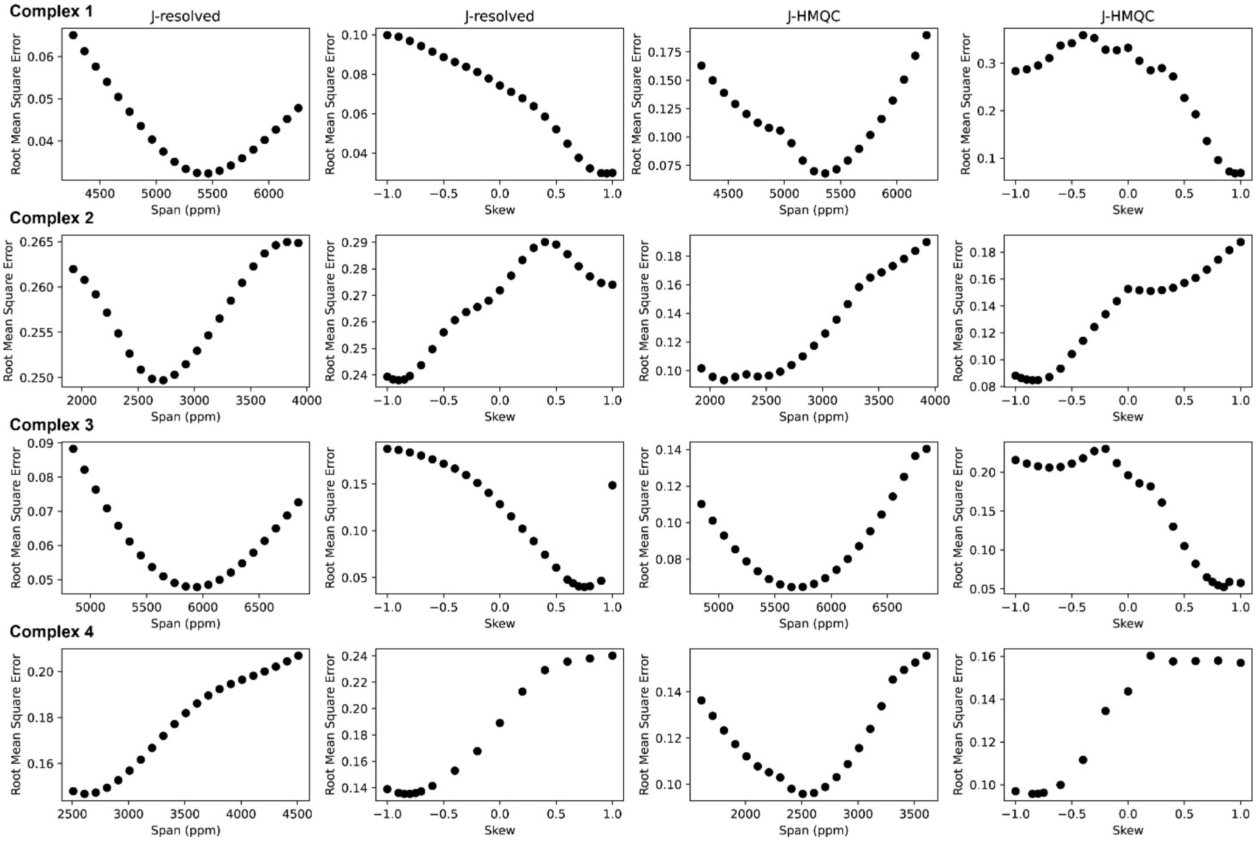

Figure S3: Root mean square error plots of the span (Ω) and skew (κ) for the *J*-resolved and *J*-HMQC sideband selective experiments on **1-4**.

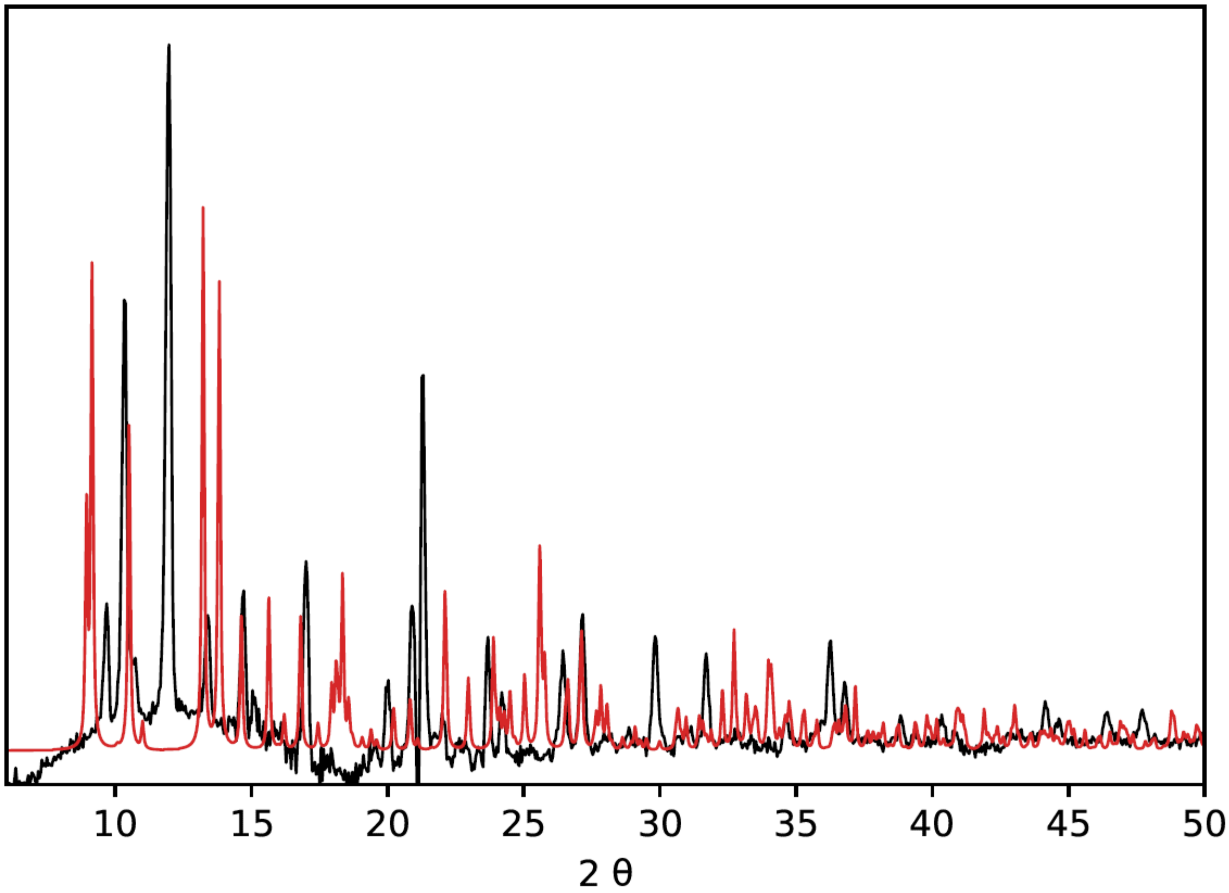

Figure S4: Experimental powder X-ray diffraction pattern of **3** (black). The pattern calculated from the single crystal X-ray diffraction structure of **3** is also shown (red). The disagreement between the two patterns occurs because the compound has lost the solvated CH_2Cl_2 which was incorporated into the lattice.

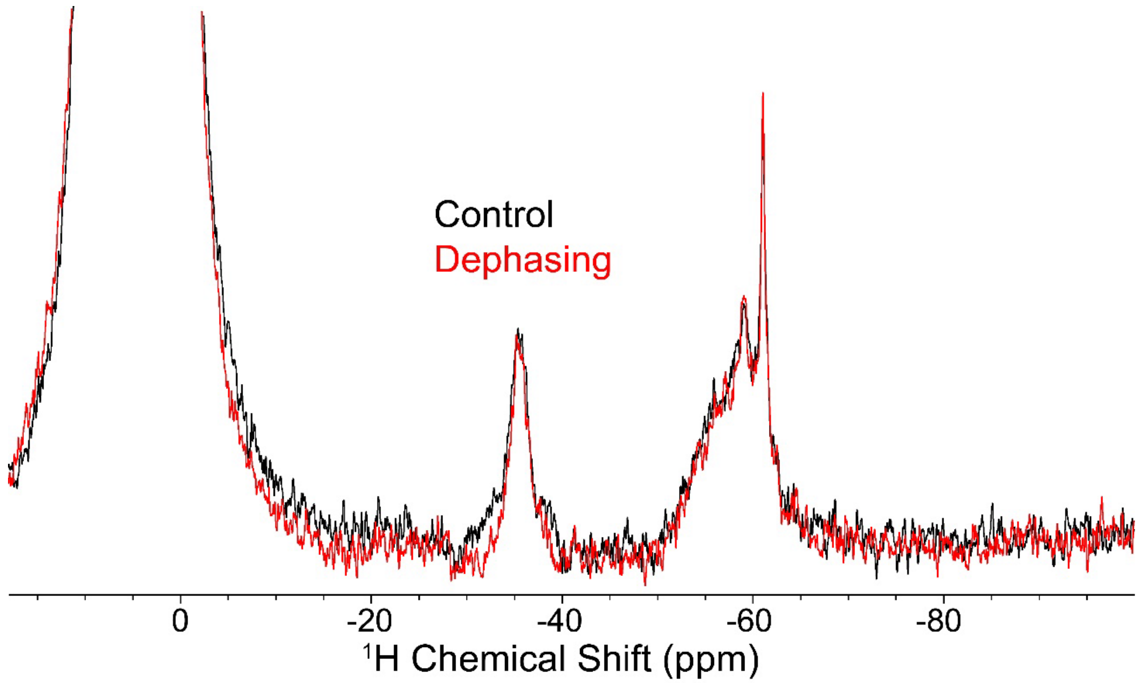

Figure S5: $^{1}H\{^{195}Pt\}$ *J*-resolved control and dephasing spectra for **1/SiO_2-Al_2O_3** acquired at room temperature and 25 kHz MAS.

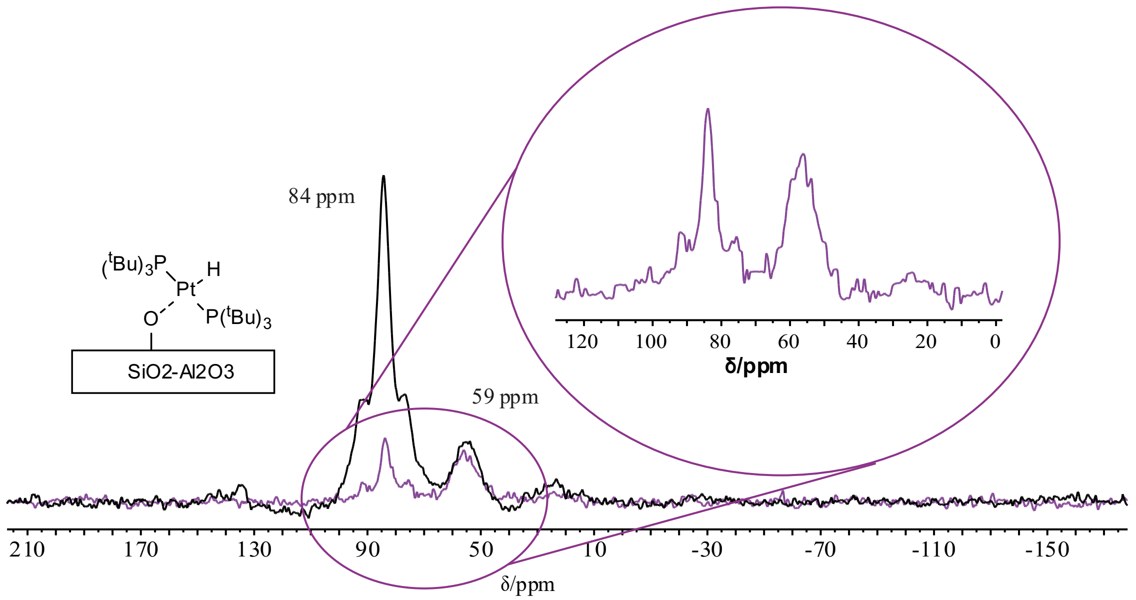

Figure S6: ^{31}P CPMAS NMR control experiment on **1/SiO$_2$-Al$_2$O$_3$** impregnated with TEMPO dissolved in TCE (magenta) and without impregnation (grey). All spectra were recorded at room temperature.

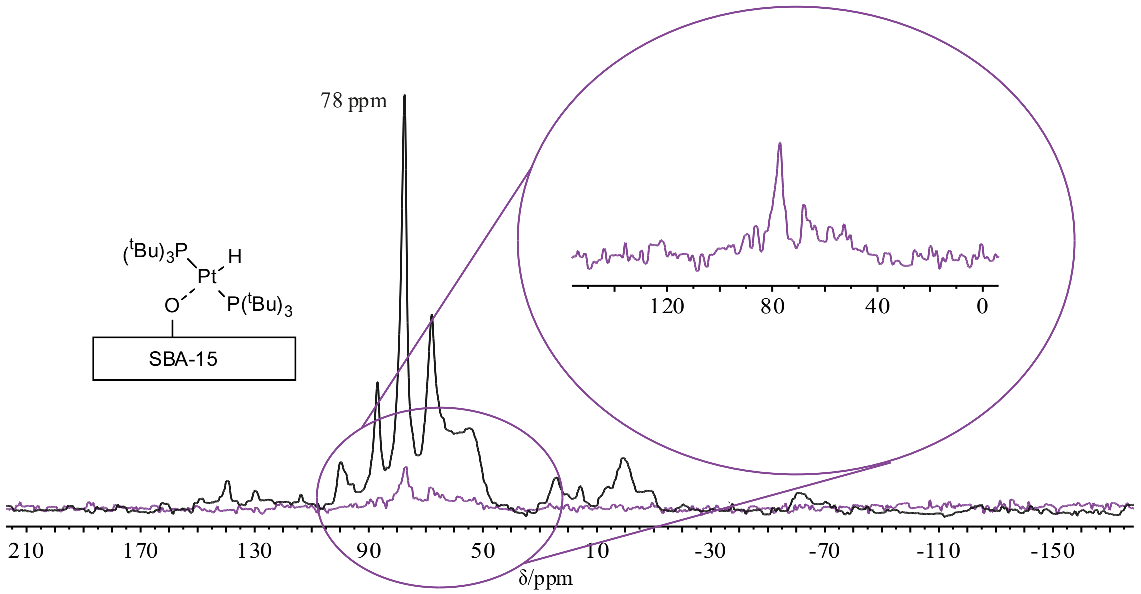

Figure S7: ^{31}P CPMAS NMR control experiment on **1/SBA-15** impregnated with TEMPO dissolved in TCE (magenta) and without impregnation (grey).

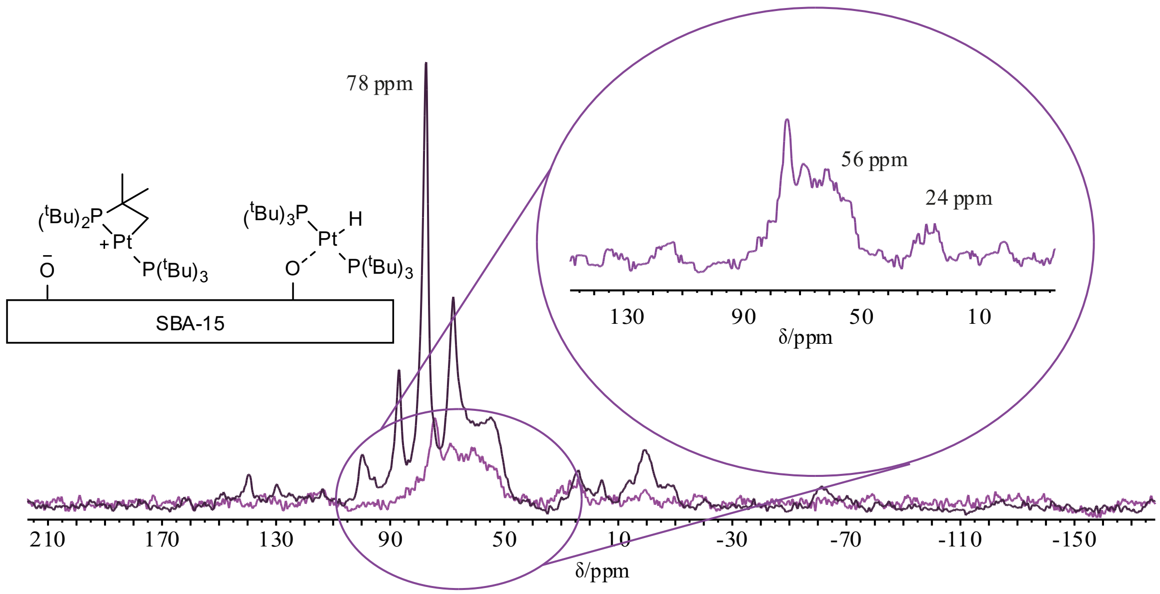

Figure S8: ^{31}P CPMAS ssNMR spectra of **1/SBA-15**, freshly prepared (grey) and >1 year old (magenta).

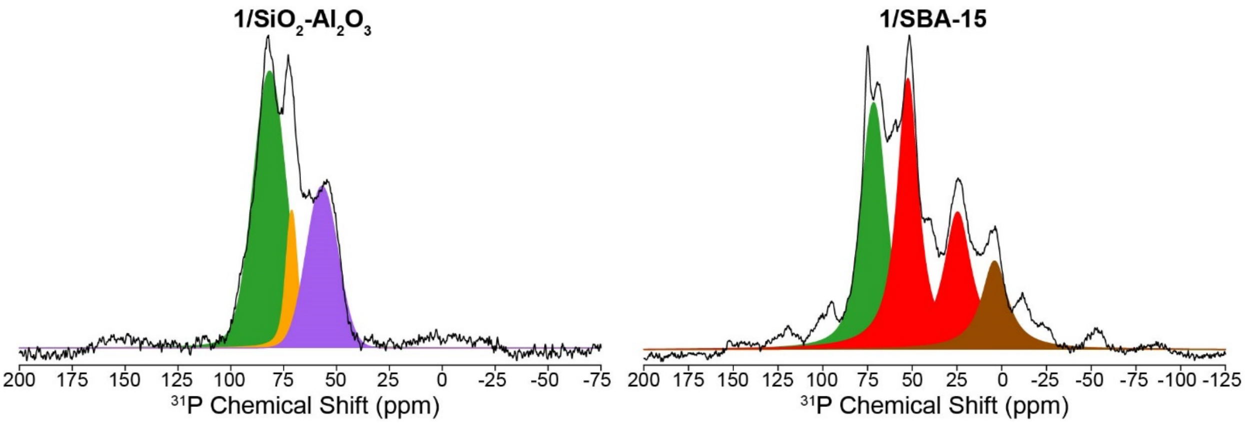

Figure S9: Peak fits of the DNP-enhanced ^{1}H-^{31}P CPMAS spin echo ssNMR spectra of **1/SiO_2-Al_2O_3** and **1/SBA-15.** The fits were conducted in ssNake.

Table S1: Chemical shifts and relative integration for the ^{31}P sites of the two surface-supported complexes

**1/SiO_2-Al_2O_3**		
**Site**	**^{31}P Chemical Shift (ppm)**	**Relative Integration from Analytical Fit**
**Hydride (Green)**	81.67	57.66%
**Phosphine Oxide (Orange)**	71.23	11.70%
**Protonated Phosphine (Purple)**	56.88	30.64%
**1/SBA-15**		
**Site**	**^{31}P Chemical Shift (ppm)**	**Relative Integration from Analytical Fit**
**Hydride (Green)**	71.57	30.91%
**C-H Activated (Red)**	52.44	32.88%
**C-H Activated (Red)**	24.66	22.55%
**Oxidized Decomposition (Brown)**	4.000	13.66%

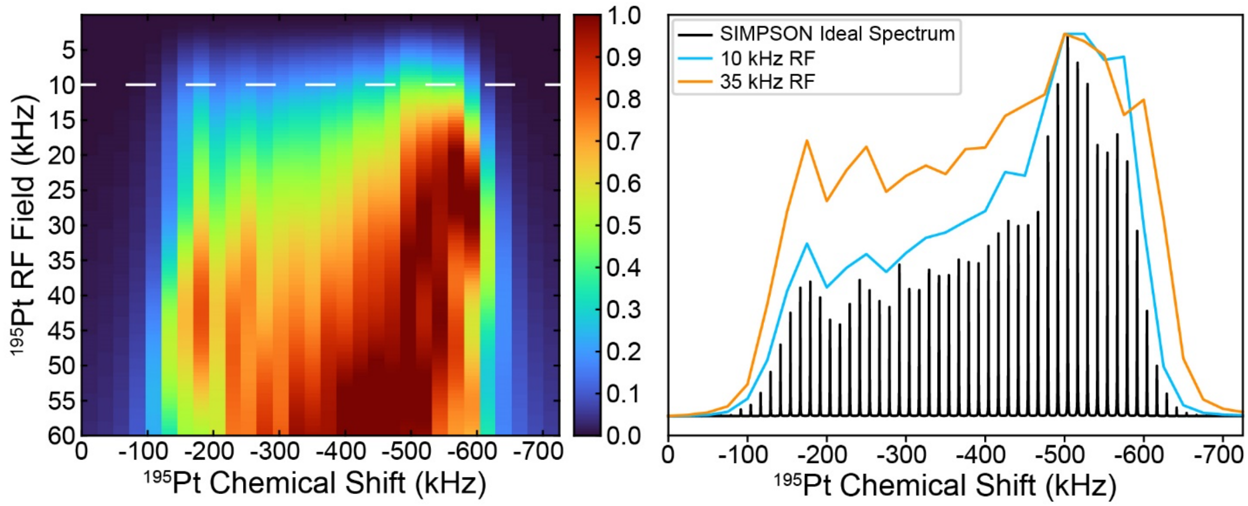

Figure S10: (Left) SIMPSON calculated ^{31}P signal dephasing for ^{1}H-$^{31}P\{^{195}Pt\}$ *J*-resolved sideband selective experiments as a function of the ^{195}Pt saturation radiofrequency (RF) field for complex **1/SiO_2-Al_2O_3**. A white dashed line indicates the optimal RF. Simulations used a 12.5 kHz spinning frequency and 120 µs saturation pulses. (Right) Graph of the SIMPSON calculated ideal ^{195}Pt ssNMR spectrum of **1/SiO_2-Al_2O_3** (black) along with simulated ^{1}H-$^{31}P\{^{195}Pt\}$ *J*-resolved sideband selective experiments that used 10 kHz (blue) and 35 kHz (orange) ^{195}Pt saturation RF fields.

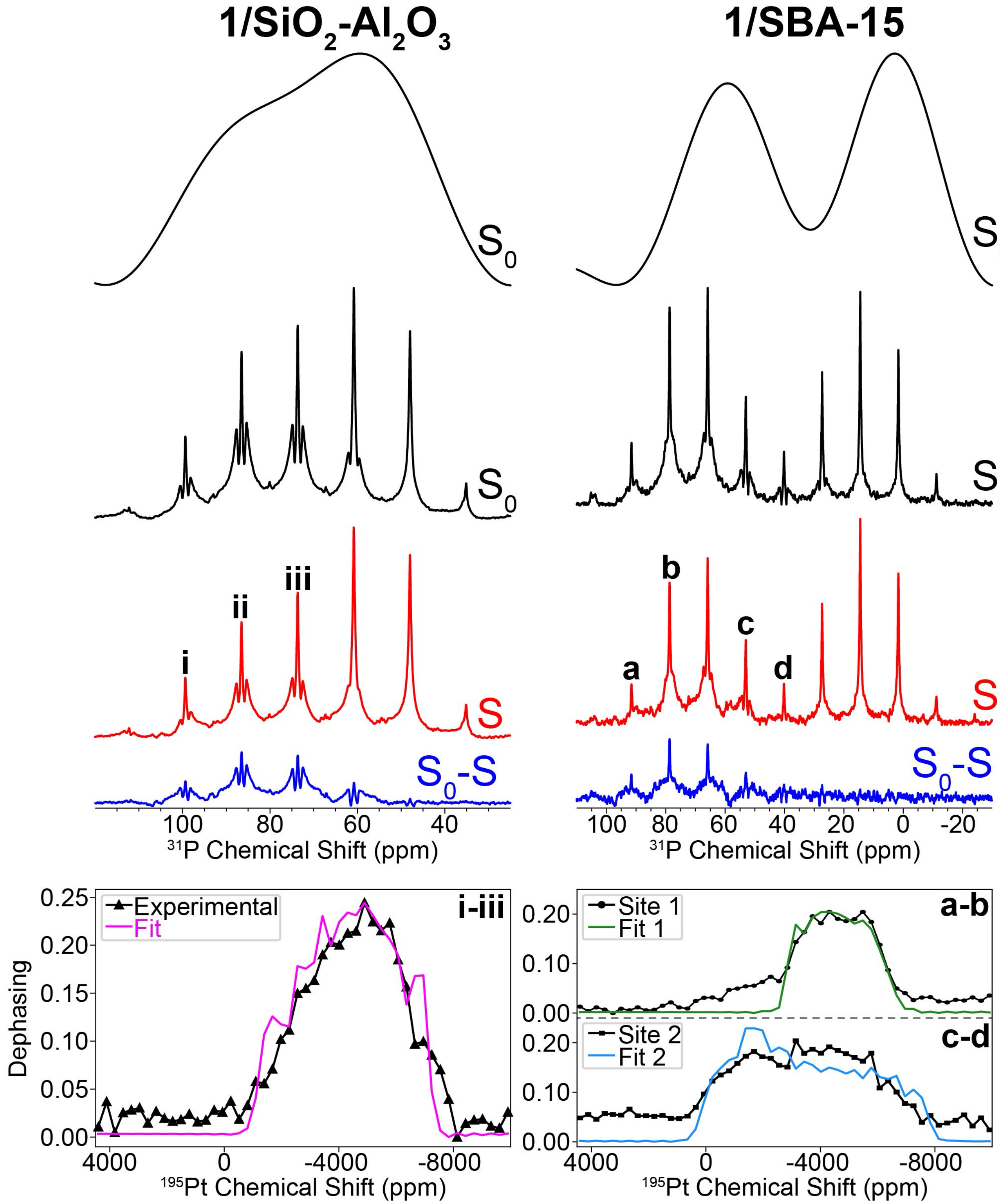

Figure S11: $^{31}P\{^{195}Pt\}$ S_0, S, and S_0–S *J*-resolved ssNMR spectra showing the CPMG spikelets that were used to construct the ^{195}Pt spectra. The co-added spectra are shown above the CPMG spikelets. For **1/SBA-15** the ^{195}Pt dephasing profile for site 1 was obtained by plotting combined dephasing for spikelets a and b, while site 2 was obtained by plotting the combined dephasing for spikelets c and d.

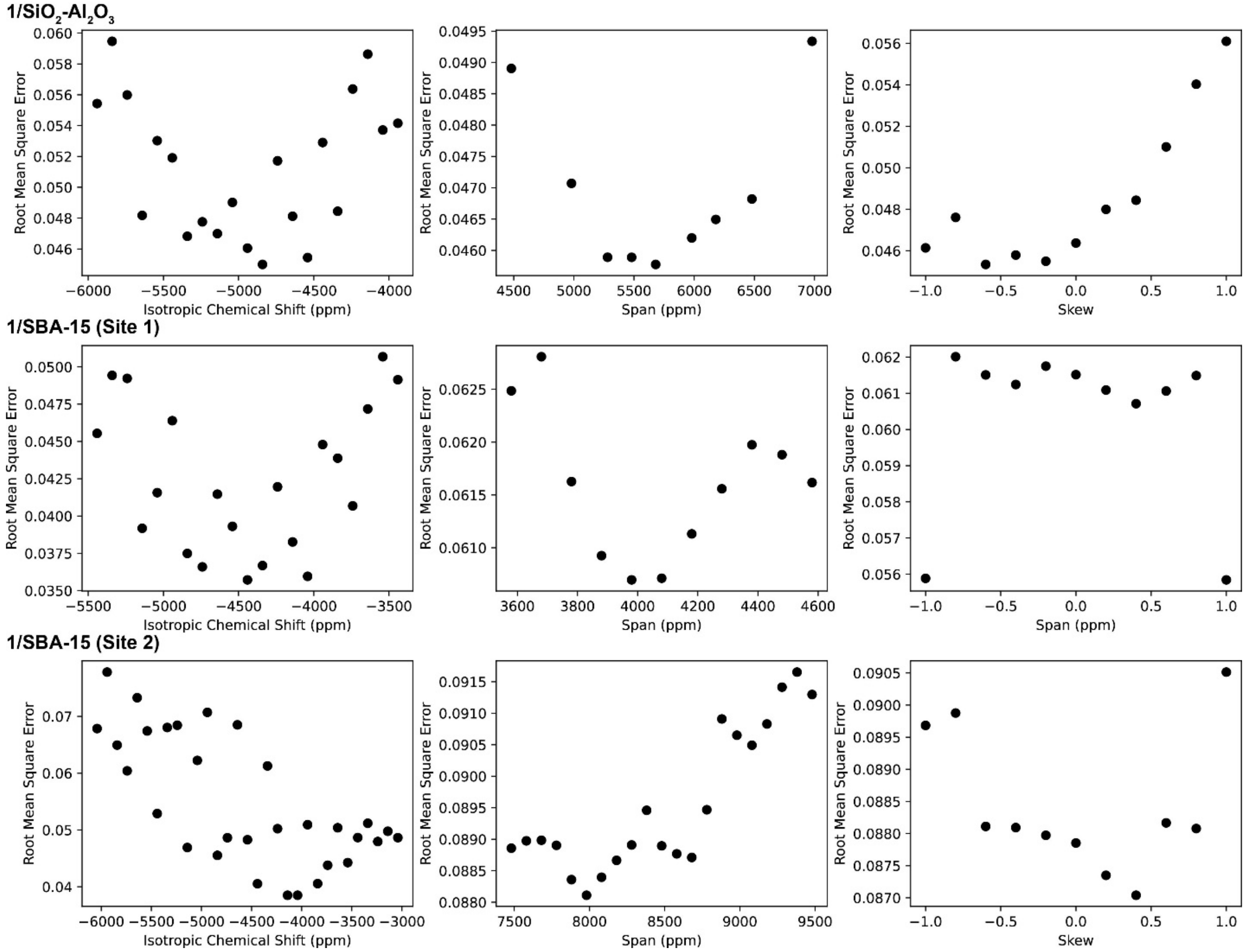

Figure S12: Root mean square error plots of the isotropic chemical shift (δ_{iso}), span (Ω), and skew (κ) for the *J*-resolved experiments on **1/SiO$_2$-Al$_2$O$_3$** and **1/SBA-15**.

Table S2: Calculated and experimental chemical shifts for the hydride and phosphine ligands.

**1/SBA-15**				
**Model**	**^{1}H Chemical Shift (ppm)**	**^{31}P Chemical Shift (ppm)**	**^{1}H-^{195}Pt *J*-coupling (Hz)**	**^{31}P-^{195}Pt *J*-coupling (Hz)**
**Experiment**	-26	74	1700	2908
**Model I**	-	96	-	4811
**Model II**	-	105	-	5321
**Model III**	-23	94	1001	3375
**Model IV**	-26	99	1213	3315
**Model V**	-28	102	1682	3287
**Model VI**	-31	104	1851	3297
**Model VII**	-33	107	2086	3281
**Model VIII**	-34	98	2505	3036
**Model IX**	-37	86	3067	2908
**1/SiO_2-Al_2O_3**				
**Model**	**^{1}H Chemical Shift (ppm)**	**^{31}P Chemical Shift (ppm)**	**^{1}H-^{195}Pt *J*-coupling (Hz)**	**^{31}P-^{195}Pt *J*-coupling (Hz)**
**Experiment**	-35	74	2400	2433
**Model A**	-	90	-	4917
**Model B**	-	115	-	7560
**Model C**	-29	81	1294	3595
**Model D**	-30	84	1477	3571
**Model E**	-32	85	1882	3434
**Model F**	-35	84	2247	3271

For all the following crystal structures, the atoms are color coded in the following scheme: C = dark gray, H = white, Pt = light gray, P = orange, Cl = green, B = pink, and F = yellow.

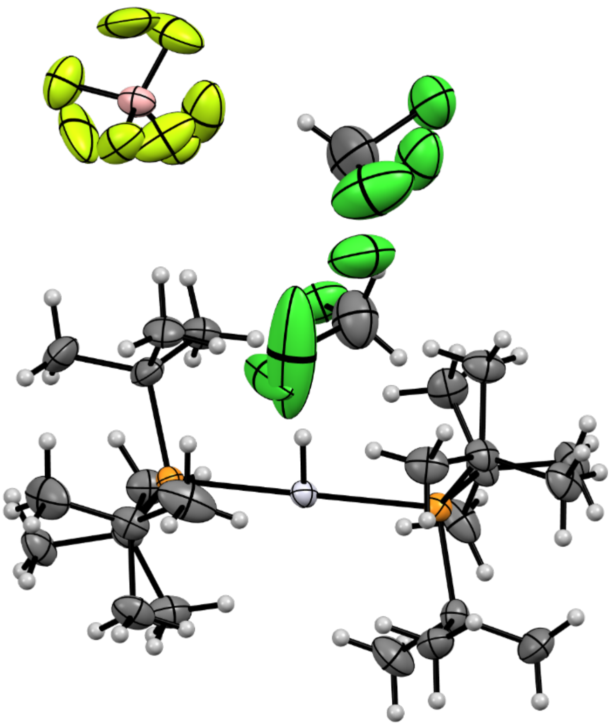

Figure S13: Thermal-ellipsoid diagram of the asymmetric unit of Pt-H(P^{t}Bu$_3$)$_2$[BF$_4$] · 2CH$_2$Cl$_2$ **3.** The DCM and BF_4^- positions in the crystal structure are disordered for to two different reasons. While BF_4^- is tetrahedral, resulting in rotational disorder, and CH_2Cl_2 is a solvent that adds in free spaces, it shows vibrational disorder from the C-Cl vibration.

Table S3: X-ray diffraction parameters of **3**.

Parameter	Pt-H(PtBu$_3$)$_2$ [BF$_4$] · 2CH$_2$Cl$_2$ (**3**)
Chemical Formula	$C_{26}H_{59}BC_{14}F_4P_2Pt$
Crystal size /mm^3	0.242 x 0.198 x 0.124
Crystal system, Space group	Monoclinic, Cc
Volume /Å^3	3712.06(18)
a /Å	11.1587(3)
b /Å	19.7667(5)
c /Å	16.9439(5)
α /°	90
β /°	96.6660(10)
γ /°	90
Z	4
Formula weight /Da	857.37
μ /mm^{-1}	4.190
F(000)	1728
Temperature /K	150(2)
Total reflections	31125
Unique reflections	9210 [R_{int} = 0.0259]
Final R indices (I > 2σ(I))	R1 = 0.0239, wR2 = 0.0512
Largest diff. peak / hole	1.089 / -0.839
GOF	1.033

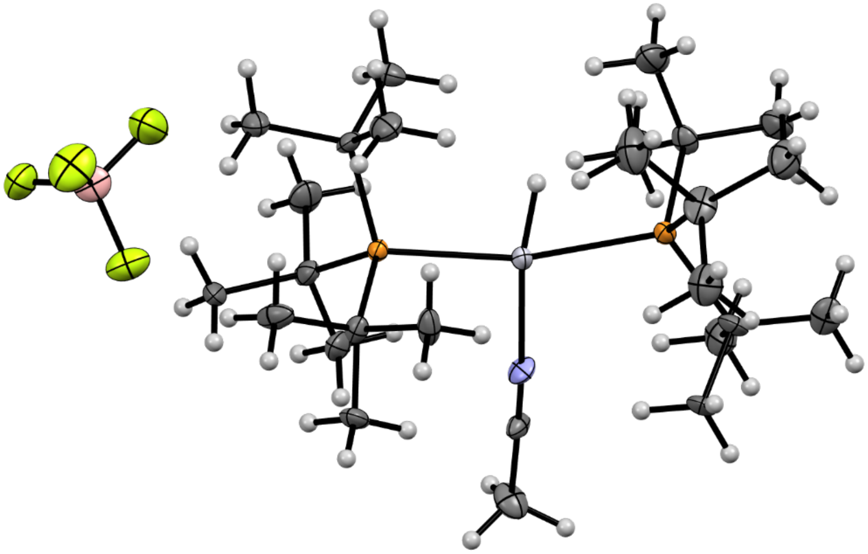

Figure S14: Thermal-ellipsoid diagram of the asymmetric unit of Pt-H(P^tBu_3)$_2$(CH_3CN)[BF_4] **4.**

Table S4: X-ray diffraction parameters of **4**.

Parameter	Pt-H(PtBu$_3$)$_2$(CH_3CN) [BF_4] (**4**)
Chemical Formula	$C_{26}H_{58}BF_4NP_2Pt$
Crystal size /mm^3	0.073 x 0.062 x 0.043
Crystal system, Space group	Monoclinic, P2(1)/n
Volume /Å^3	3095.2(14)
a /Å	8.585(2)
b /Å	12.110(3)
c /Å	29.773(6)
α /°	90
β /°	96.461(9)
γ /°	90
Z	4
Formula weight /Da	728.57
μ /mm^{-1}	4.677
F(000)	1480
Temperature /K	150(2)
Total reflections	28228
Unique reflections	7714 [R_{int} = 0.1200]
Final R indices (I > 2σ(I))	R1 = 0.0863, wR2 = 0.1705
Largest diff. peak / hole	5.440 / -3.319
GOF	1.046

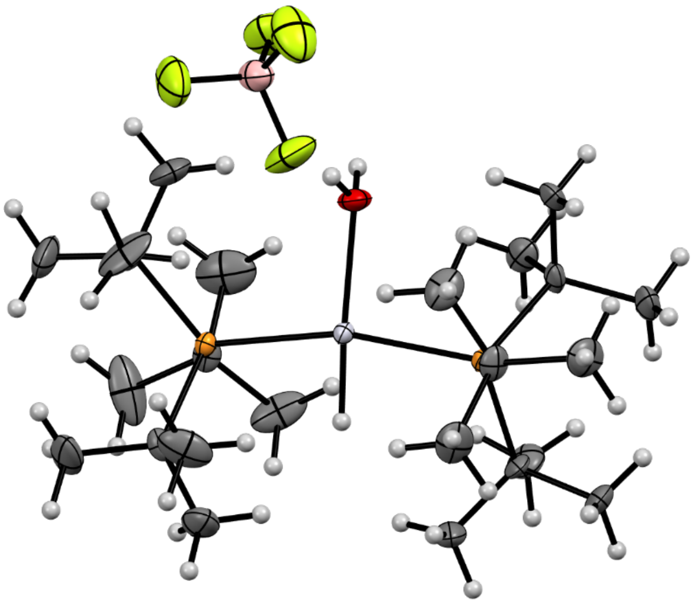

Figure S15: Thermal-ellipsoid diagram of the asymmetric unit of Pt-H(P^{t}Bu$_3$)$_2$(H$_2$O)[BF$_4$] **5.**

Table S5: X-ray diffraction parameters of **5**.

Parameter	Pt-H(P^{t}Bu$_3$)$_2$(H$_2$O)[BF$_4$] (**5**)
Chemical Formula	$C_{24}H_{57}BF_4OP_2Pt$
Crystal size /mm^3	0.143 x 0.099 x 0.074
Crystal system, Space group	Monoclinic, P2(1)/c
Volume /Å^3	3032.62(16)
a /Å	11.5231(4)
b /Å	16.5896(4)
c /Å	16.5713(5)
α /°	90
β /°	106.800(2)
γ /°	90
Z	4
Formula weight /Da	705.53
μ /mm^{-1}	4.772
F(000)	1432
Temperature /K	150(2)
Total reflections	28688
Unique reflections	7528 [R_{int} = 0.0787]
Final R indices (I > 2σ(I))	R1 = 0.0412, wR2 = 0.0523
Largest diff. peak / hole	1.218 / -1.158
GOF	1.014

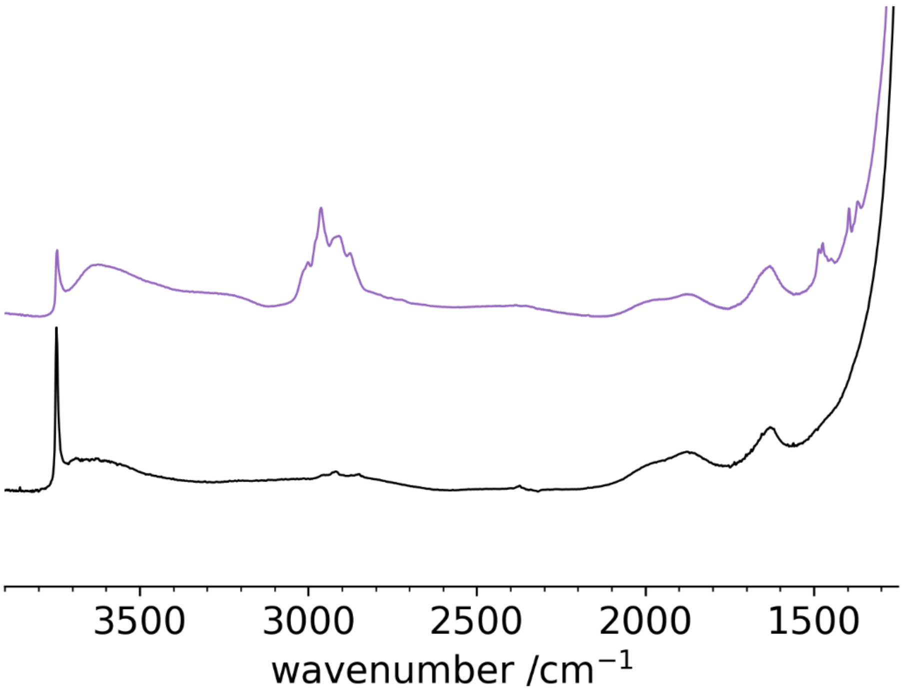

Figure S16: IR-spectrum of SiO_2-Al_2O_3-500 dehydroxylated at 500°C (bottom, black) and Pt-H@SiO_2-Al_2O_3-500 (top, purple).

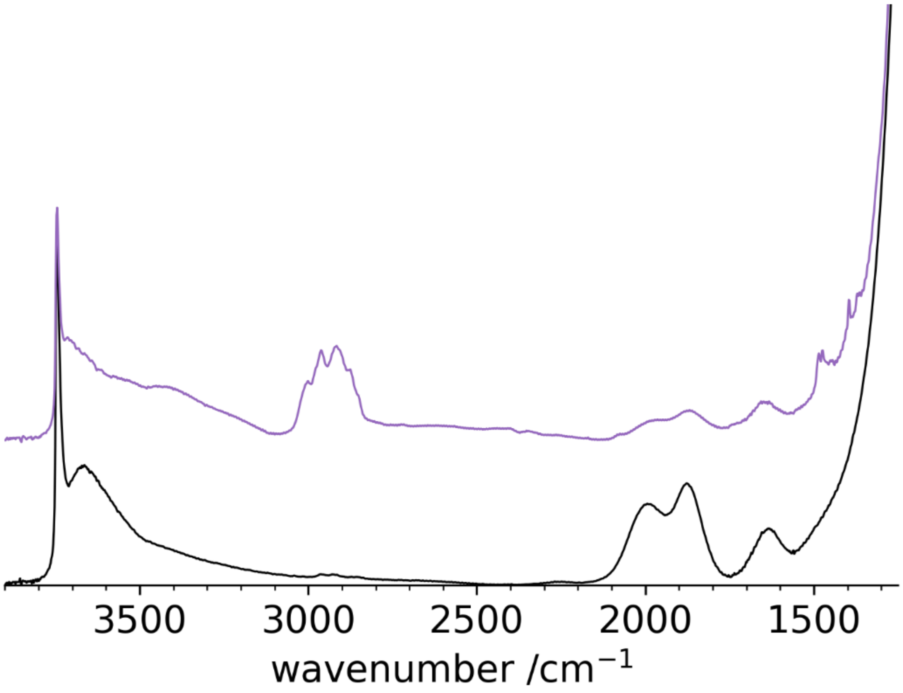

Figure S17: IR-spectrum of SBA-15-500 dehydroxylated at 500°C (bottom, black) and Pt-H@Silica-500 (top, purple).

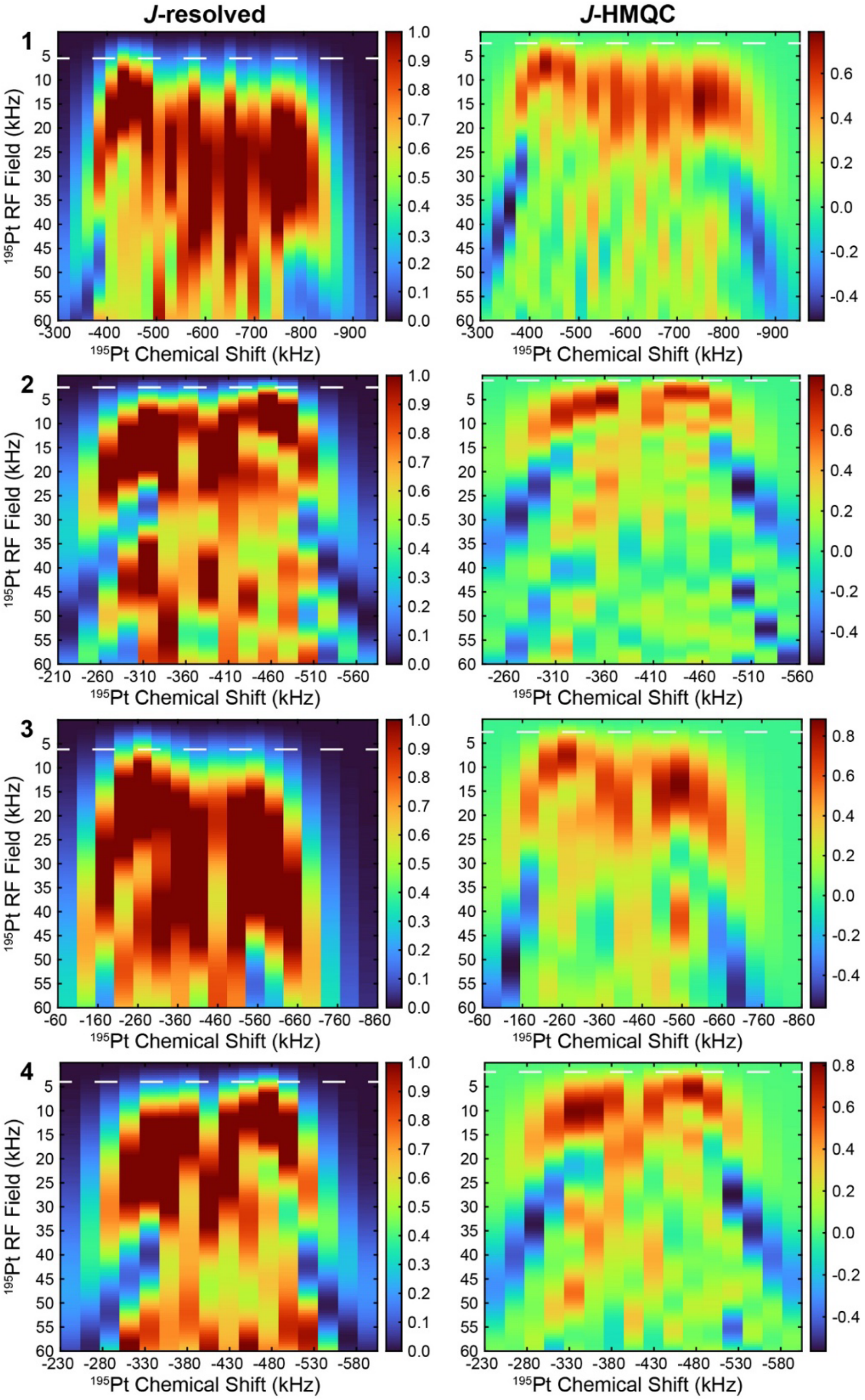

Figure S18: SIMPSON calculated ^{31}P signal dephasing or efficiency for ^{1}H-$^{31}P\{^{195}Pt\}$ and $^{1}H\{^{195}Pt\}$ *J*-resolved and *J*-HMQC sideband selective experiments, respectively, as a function of the ^{195}Pt saturation radiofrequency (RF) field. Heat maps are labeled **1** through **4**, corresponding to molecular complexes **1** through **4**. Simulations for **1** used a 25 kHz spinning frequency and 80 µs saturation pulses, **2** used a 25 kHz spinning frequency and 120 µs saturation pulses, **3** used a 50 kHz spinning frequency and 60 µs saturation pulses, and **4** used a 25 kHz spinning frequency and 80 µs saturation pulses. A white dashed line indicates the optimal RF field for all heat maps.

Table S6: Optimal ^{195}Pt saturation RF fields for the molecular precursors

Complex	***J*-resolved (kHz)**	***J*-HMQC (kHz)**
**1**	6	3
**2**	3	2
**3**	7	3
**4**	4	2
